# Assessing Geographical Origin of *Gentiana Rigescens* Using Untargeted Chromatographic Fingerprint, Data Fusion and Chemometrics

**DOI:** 10.3390/molecules24142562

**Published:** 2019-07-14

**Authors:** Tao Shen, Hong Yu, Yuan-Zhong Wang

**Affiliations:** 1 Yunnan Herbal Laboratory, Institute of Herb Biotic Resources, School of Life and Sciences, Yunnan University, Kunming 650091, China; 2 The International Joint Research Center for Sustainable Utilization of Cordyceps Bioresouces in China and Southeast Asia, Yunnan University, Kunming 650091, China; 3 College of Chemistry, Biological and Environment, Yuxi Normal University, Yu’xi 653100, China; 4 College of Traditional Chinese Medicine, Yunnan University of Chinese Medicine, Kunming 650500, China

**Keywords:** authentication, liquid chromatography fingerprint, chemometrics, random forest, OPLS-DA, data fusion, *Gentiana rigescens*

## Abstract

*Gentiana rigescens* Franchet, which is famous for its bitter properties, is a traditional drug of chronic hepatitis and important raw materials for the pharmaceutical industry in China. In the study, high-performance liquid chromatography (HPLC), coupled with diode array detector (DAD) and chemometrics, were used to investigate the chemical geographical variation of *G. rigescens* and to classify medicinal materials, according to their grown latitudes. The chromatographic fingerprints of 280 individuals and 840 samples from rhizomes, stems, and leaves of four different latitude areas were recorded and analyzed for tracing the geographical origin of medicinal materials. At first, HPLC fingerprints of underground and aerial parts were generated while using reversed-phase liquid chromatography. After the preliminary data exploration, two supervised pattern recognition techniques, random forest (RF) and orthogonal partial least-squares discriminant analysis (OPLS-DA), were applied to the three HPLC fingerprint data sets of rhizomes, stems, and leaves, respectively. Furthermore, fingerprint data sets of aerial and underground parts were separately processed and joined while using two data fusion strategies (“low-level” and “mid-level”). The results showed that classification models that are based OPLS-DA were more efficient than RF models. The classification models using low-level data fusion method built showed considerably good recognition and prediction abilities (the accuracy is higher than 99% and sensibility, specificity, Matthews correlation coefficient, and efficiency range from 0.95 to 1.00). Low-level data fusion strategy combined with OPLS-DA could provide the best discrimination result. In summary, this study explored the latitude variation of phytochemical of *G. rigescens* and developed a reliable and accurate identification method for *G. rigescens* that were grown at different latitudes based on untargeted HPLC fingerprint, data fusion, and chemometrics. The study results are meaningful for authentication and the quality control of Chinese medicinal materials.

## 1. Introduction

*Gentiana rigescens* Franchet (Dian long dan) is a herbaceous species that grows in mountainous regions of Yunnan-Guizhou Plateau in the southwest of China [1]. Like European traditional medicinal plant yellow gentian (*G. lutea* L), *G. rigescens* is famous for its bitter properties that are due to the bitter active principles (e.g., loganin, gentiopicroside, swertiamarin, sweroside, etc.) [2,3,4]. Those compounds have pharmacological effects of anti-inflammation, antioxidant, anti-cancer, antiviral, cholagogic agent, hepatoprotective, wound-healing activities, and so forth [3,5]. Additionally, they are used to stimulate appetite and improve digestion [5,6,7]. In addition, a series of neuritogenic compounds had been isolated from the aerial and underground parts of *G. rigescens*, which could be used as raw material for the preparation of functional food and a therapeutic drug for Alzheimer’s disease [8,9,10,11]. Now, *G. rigescens* have been the official drug of Chinese pharmacopoeia (2015 edition) for chronic hepatitis and important raw materials for the pharmaceutical industry in China [12].

*G. rigescens* were usually collected from different regions of Yunnan-Guizhou Plateau in order to provide satisfaction of continuously increasing industrial demands for raw materials. However, some of the researchers had reported that chemical constitutions of underground part of *G. rigescens* were extremely variable and diverse according to plant grown location or producing area [13,14,15]. Quantitative analysis of bioactivity compounds (such as gentiopicroside, sweroside, swertiamarin, isoorientin, and other compounds) from rhizomes, stems, leaves, and flowers indicated that northwest of Yunnan-Guizhou Plateau was suitable for chemical compounds accumulation [13,14,15,16]. Additionally, conversion and transport of those compounds might be influenced by climatic conditions in the plant habitat [14,17].

Latitude has a strong impact on the local climate environment in southwest China [18,19]. As the main distribution area of *G. rigescens*, Yunnan-Guizhou Plateau is characterized by very complex topography and it displays a wide variety of micro-climates [18,19,20,21]. There are six climatic zones from the north towards the south [20]. Especially, in the higher latitude areas, such as northwest Yunnan or south of the Hengduan Mountains (26–28° N), the temperature gradients are more abrupt than in the other regions [19]. Furthermore, precipitation and temperature in the Yunnan-Guizhou Plateau also show clear variations along the latitude gradients [19,21]. Therefore, it is necessary to explore the variation of phytochemical and medicinal material quality of *G. rigescens* that were grown in different latitudes and build a classification model for tracing producing areas of medicinal materials.

As we know, the contents of bioactive compounds and quality of medicinal materials have a close relationship with the environment of producing area [22,23,24,25]. Quality control and geographical indication of medicinal materials raise many concerns by pharmaceutical industries with the expansion in the use of herbal medicines. However, using few marker compounds could not reflect the chemical complexity of herbs and this method is hard to effectively authenticate the origin of herbal medicines [26,27]. Chemical fingerprints, as a comprehensive evaluation methodology, have been widely used to deal with the problem [26,28,29]. In recent years, infrared spectroscopy (IR), UV-Vis spectroscopy (UV-Vis), and other spectral fingerprints have been well-established analytical techniques for geographical traceability studies of *G. rigescens* and other medicinal plants in the worldwide [30,31,32,33,34]. In contrast, there were limited reports on the use of chromatographic fingerprint to identify the producing regions of herbal materials [30,31,32,33,34,35]. Although there were many reports about discrimination of herbs according to their producing areas while using liquid chromatography technology, most of them are based on the information of limited chemical markers or chromatographic profiles [36,37,38,39]. The potential of chromatographic fingerprints for herbs authentication needs to be further explored.

When compared with chemical marker or chromatographic profile (targeted), chromatographic fingerprint (untargeted) contains unspecific and non-evident information and chemometric tools should extract chemical information [40]. Recently, literature reported some successful studies applying chromatographic fingerprint, together with chemometric methodology, to discriminate herbs and food samples of different origin or cultivars [41,42,43,44]. All of those studies suggested that it is possible to develop a reliable and accurate method for the geographical tracing of *G. rigescens* by applying the chromatographic fingerprint methodology.

In the progression of improving geographical authentication of food and drugs, one of the important goals is building discrimination models with a less error rate and reducing the uncertainty of the prediction results [33,44]. Data fusion strategy has been widely used in the last years in the field of food authentication in order to improve class discrimination techniques [45]. Some reports about *Panax notoginseng*, *Paris Polyphylla* var. *yunnanensis* and other herb materials also showed the huge potential of this strategy in the discrimination of medicinal materials producing areas [46,47,48]. Today, most of the fused data come from spectral fingerprint and very few studies report the data fusion of chromatographic fingerprint [42,43]. Furthermore, data fusion studies are mostly based on the fusion of multivariate instrumental techniques [42,43], while reports of *P. Polyphylla* var. *yunnanensis*, *Macrohyporia cocos*, and other species indicated that reliable classification results were also available by the fusion analysis of chemical fingerprint data collected from different medicinal parts of herbs [35,49]. Accumulation and distribution of metabolites in the different parts of plants were different because of the differential response of root, stem, flower and other organs to the environment variation of producing area [17,50]. Therefore, fingerprint data fusion of multi-medicinal parts may provide integrated chemical information for the authentication of medicinal materials. At the same time, this method also contributes to a more comprehensive understanding of the response and adaptation of medicinal plants to complex geographical environments.

The aim of this study is to explore the variation of chromatographic fingerprints of *G. rigescens* along the latitude gradients and to use chemometrics to mine fingerprint chemical information, and to investigate the potential of the untargeted chromatographic fingerprint to trace herbs grown at different latitudes. For this purpose, we developed fingerprint of rhizomes, stems, and leaves of *G. rigescens* by high-performance liquid chromatography with diode array detection (HPLC-DAD) technology. Subsequently, classification models for the identification of different producing areas were built by HPLC fingerprint combined with RF (random forest algorithm) and OPLS-DA (orthogonal partial least-squares discriminant analysis). At last, two types of data fusion strategies, “low- level” and “mid-level” data fusion, were studied in order to improve the model performances.

## 2. Results and Discussion

### 2.1. Chromatographic Fingerprints Variation Along the Latitude Gradients

**Figure 1:**
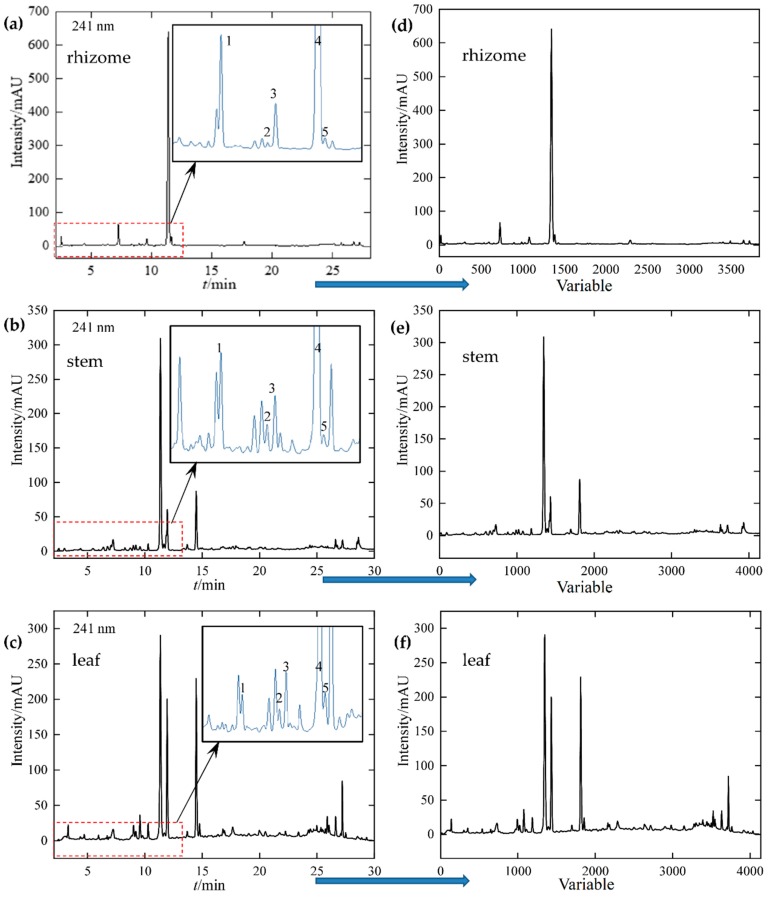
High-performance liquid chromatography (HPLC) fingerprint of rhizome (**a**), stem (**b**), leaf (**c**) and fingerprints after variable transformation (**d**–**f**). (1) loganin, (2) 6′-*O*-β-d-glucopyranosylgentiopicroside, (3) swertiamarine, (4) gentiopicroside, and (5) sweroside.

Figure 1 displays the representative chromatographic fingerprints of rhizome, stem, and leaf. From HPLC fingerprints, it can be found that the five marker compounds of iridoids were eluted before 15 min. The retention times (*t*/min) of loganin (1), 6′-*O*-β-d-glucopyranosylgentiopicroside (2), swertiamarine (3), gentiopicroside (4), and sweroside (5) were 7.279, 9.213, 9.573, 11.376, and 11.622 min, respectively. Loganin and gentiopicroside were mainly accumulation in the underground part and sweroside accumulated more in the overground parts. Furthermore, differences in the chemical composition of rhizome, stem, and leaf can also be visually observed through chromatographic fingerprints. For facilitating subsequent data exploration and modeling analysis, the retention time of fingerprints signal was replaced by variables (Figure 1d–f). As a result, there were 3839, 4140, and 4140 variables of rhizome, stem, and leaf fingerprints, respectively.

**Figure 2:**
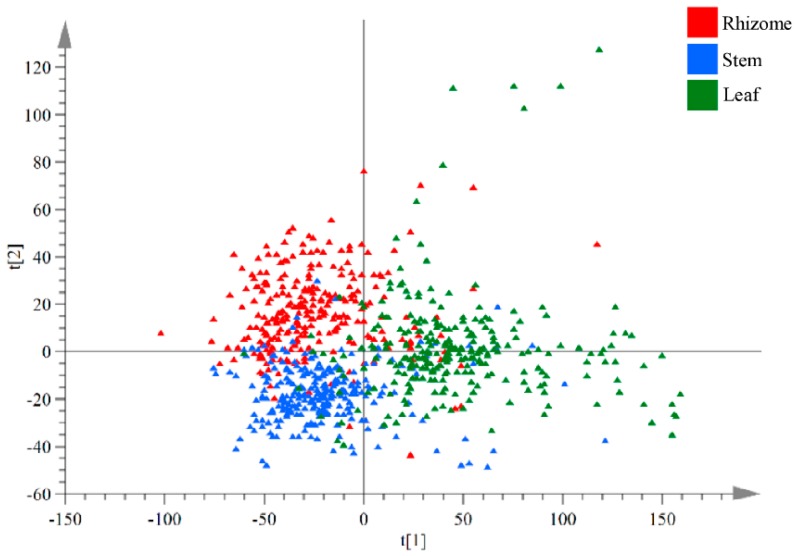
Two-dimensional principal component score plot of rhizomes, stems, and leaves samples based on chromatographic fingerprint data.

Principal component analysis (PCA) and two-dimensional score plots visualized the differences and variation trends of three medicinal parts. Figure 2 shows that the rhizomes and stems of *G. rigescens* tended to cluster to the left part, while the leaves data scattered to the right.

**Figure 3:**
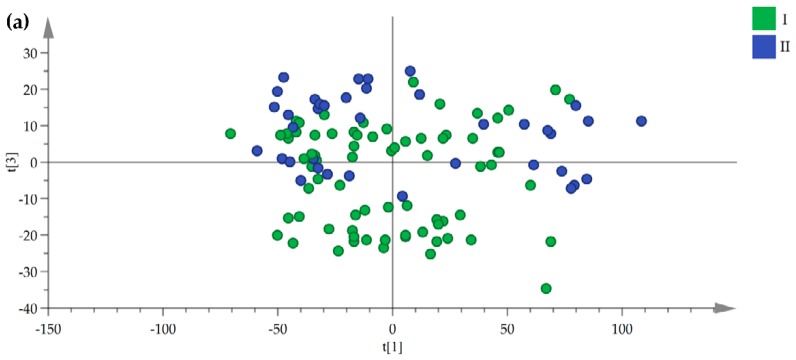

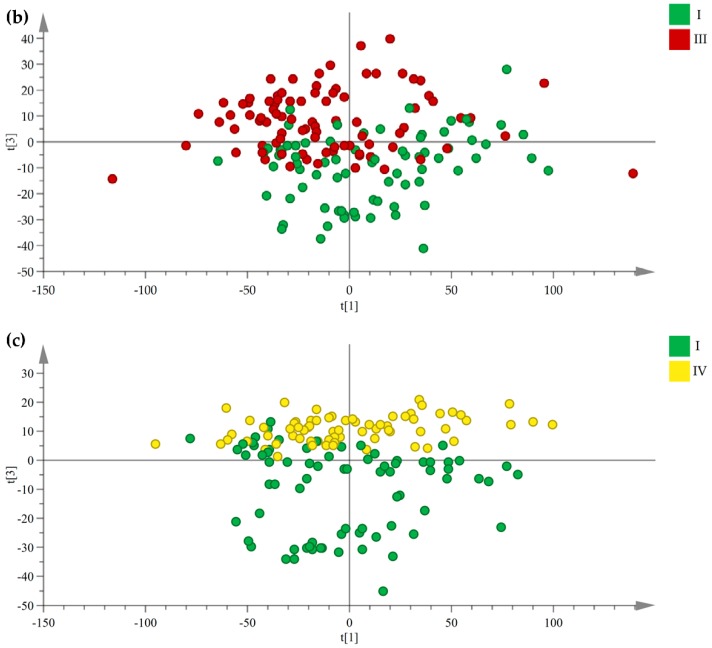
Variation of rhizomes score plots along the latitude gradients. (**a**) is low latitude and mid-latitude, (**b**) is low latitude and mid-high latitude and (**c**) is low latitude and high latitude (green circles = low latitudes area, 23.92–23.66° N, blue circles = mid-latitude area, 24.95–25.06° N, red circles = mid-high latitude area, 26.49–26.64° N, yellow circles = high latitude area, 27.34–28.52° N).

**Figure 4:**
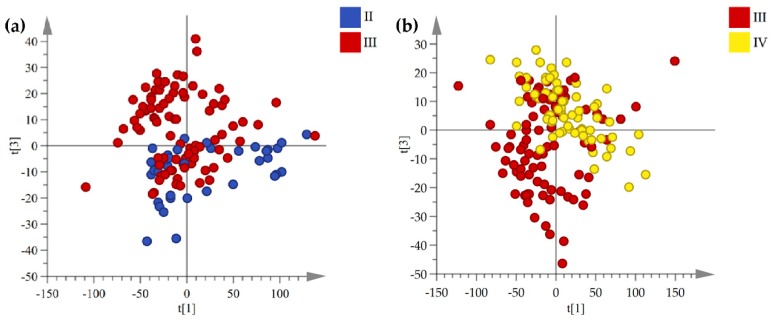
Variation of rhizomes score plots between the adjacent latitudes. (**a**) is mid-latitude and mid-high latitude and (**b**) is mid-high latitude and high- latitude (blue circles = mid-latitude area, 24.95–25.06° N, red circles = mid-high latitude area, 26.49–26.64° N, yellow circles = high latitude area, 27.34–28.52° N).

Although the fingerprints between the aboveground and underground medicinal parts were obvious differences, an interesting result is that a trend of separation according to product region latitude was observed from the PCA and score plots of samples of three medicinal parts. For example, two-dimensional score plots of chromatographic fingerprint of rhizomes showed that the samples separation trend increases with an increase in geographical distance and a clear separation between samples that were collected from lower latitude and higher latitude regions (Figure 3). In contrast to this, when considering the separation between samples with product regions geographically close to each other, we observed that the rhizome samples separation trend decreases with a decrease in the geographical distance (Figure 4). The PCA score plots of stems and leaves changed in the same trend as rhizomes (Figures S1–S4).

**Figure 5:**
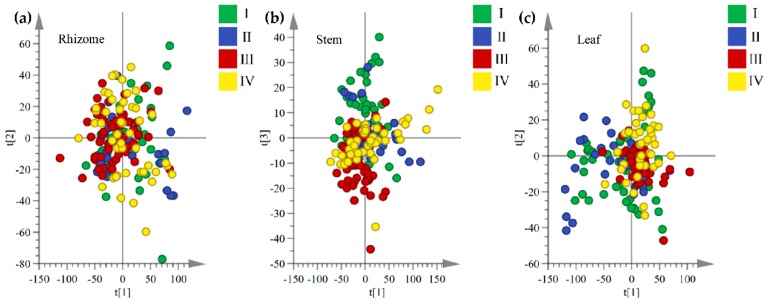
Two-dimensional principal component score plots for samples of rhizomes (**a**), stems (**b**), and leaves (**c**) of *G. rigescens* grown at four latitudes.

**Figure 6:**
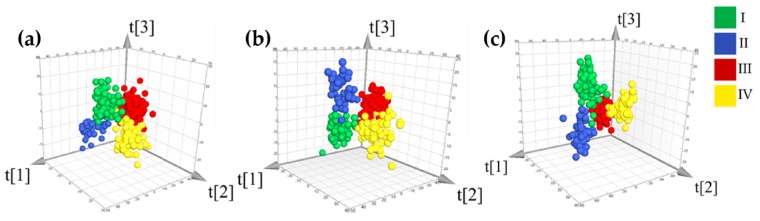
Three-dimensional (3D) Scores-plot diagram of rhizomes (**a**), stems (**b**), and leaves (**c**) orthogonal partial least-squares discriminant analysis (OPLS-DA) analysis among four different latitudes (OPLS-DA model (**a**) *R*^2^ = 0.74 and *Q*^2^ = 0.68, model (**b**) *R*^2^ = 0.75 and *Q*^2^ = 0.68, model (**c**) *R*^2^ = 0.72 and *Q*^2^ = 0.71, permutation plot of three models were shown in Figures S5–S7).

The results of PCA highlighted that the chromatographic fingerprints of *G. rigescens* were different among rhizomes, stems, and leaves, and were affected by latitude gradients of the production regions. Especially between lower latitudes and higher latitudes, the samples seem to be clearly distinguishable. Based on PCA exploratory analysis (unsupervised methods), supervised pattern recognition (OPLS-DA) should be applied to gain better classification results for samples that were grown in different latitudes (Figure 5 and Figure 6), and OPLS-DA and variable importance in the projection (VIP) analysis were used to further investigate the fingerprint variables of *G. rigescens* that were sensitive to latitude changes.

**Figure 7:**
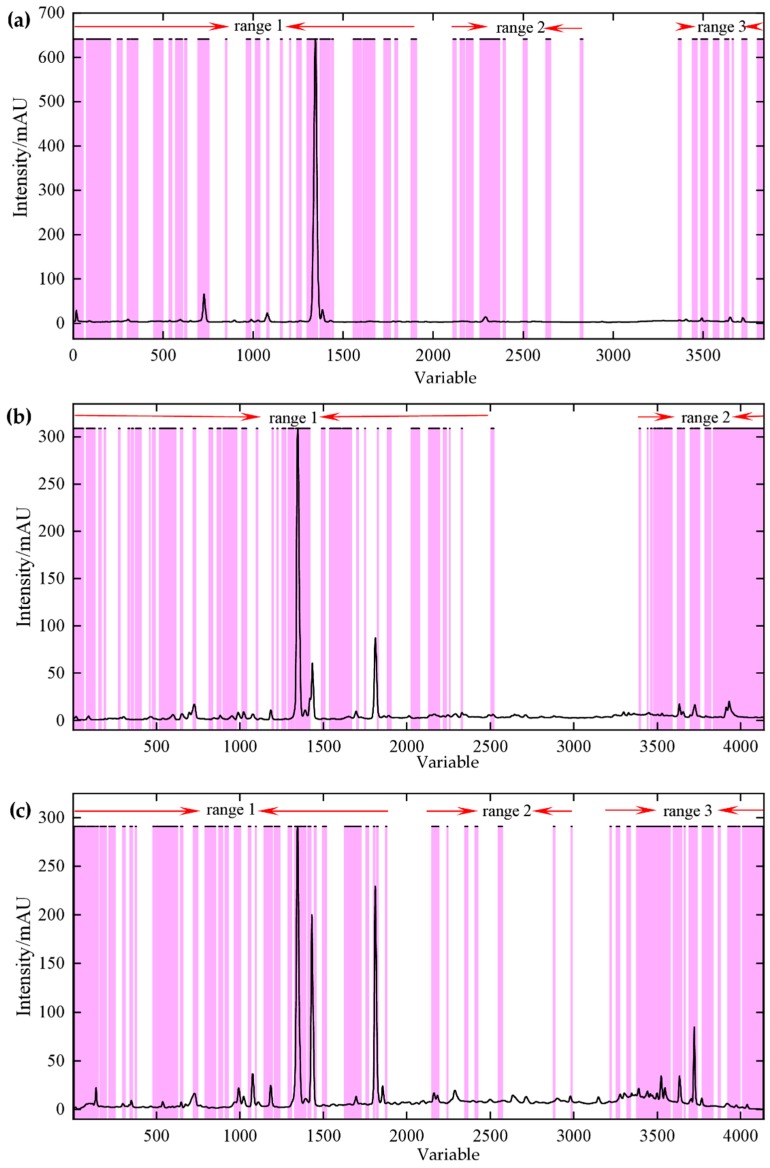
Important variables of fingerprint (purple = variable VIP value > 1) (**a**) rhizome, (**b**) stem, and (**c**) leaf.

The variable’s VIP value was greater than 1.00, which indicates that the variable was obviously affected by the change of the latitude of the producing areas. From Figure 7a, it could be found that the change of three ranges of rhizome’s fingerprint was closely related to producing areas latitude. The first range was related to variables of retention time at 2.00–13.00 min. The second range was related to variables of retention time at 15.00–20.00 min. Additionally, the third range was related to the variables of retention time after 25.00 min. Figure 7b showed that important variables (VIP value > 1.00) of stem fingerprint relate to the variables of retention time at 2.00–20.00 min and 25.00–30.00 min. For leaf fingerprint, chromatographic variables, retention time at 2.00–15.00 min, 17.00–19.00 min and 25.00–30.00 min, were the most sensitive to latitude changes of producing areas (Figure 7c). According to the identification of the major compounds in fingerprint, it showed that many of these important variables were chromatographic signals of iridoids and secoiridoids, such as loganin, 6′-*O*-β-d-glucopyranosylgentiopicroside, swertiamarine, gentiopicroside, and sweroside. A previous study regarding the spatial profiling of iridoids phytochemical constituents found that the geographical variation of those compounds could be attributed to some environmental factors [13,17], for example, the difference of precipitation of natural habitats [17]. Additionally, it was interesting to note that the number of important variables after 25 min is gradually increasing from the rhizome to the leaves. The results suggested that, in addition to iridoids, other low polarity products in *G. rigescens* have implications for the differentiation of different geographical origins.

In a word, current research indicated that the chemical composition of *G. rigescens* changes with the grown latitude in a way that could be traced with the chromatographic fingerprint. Furthermore, three-dimensional (3D) score plots and VIP analysis showed a difference of phytochemical geographic variation for overground and underground parts. Those differences might affect the result of geographical origin traceability of samples.

### 2.2. Geographic Authentication Based on Fingerprints of Different Medicinal Parts

In recent years, literature had already reported satisfying classification results that were obtained by RF or OPLS-DA models [51,52,53,54]. As an ensemble learning method, the RF algorithm could correct for decision trees’ habit of overfitting to their training set [55]. Additionally, OPLS could help to overcome these obstacles by separating useful information from noise and improve complex chemical data features and interpretability [56,57]. In this work, we tested RF and OPLS-DA models, combined with rhizome, stem, and leaf fingerprint data in order to classify *G. rigescens* according to their grown latitude.

#### 2.2.1. RF Classification

**Figure 8:**
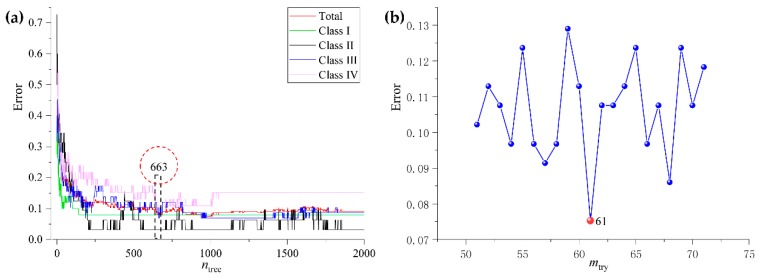
The *n*_tree_ (**a**) and *m*_try_ (**b**) screening of RF models based on rhizomes fingerprints.

In the beginning, samples from the data set of rhizomes (280 samples and 3839 variables) were separated into a calibration set (186 samples) and a validation set (94 samples) by the Kennard-Stone algorithm. Subsequently, 186 rhizome samples that were collected from four latitude gradients were used to establish the calibration model (R_RF). During the modeling process, the initial value of *n*_tree_ (needs to be optimized) was defined as 2000, the initial value of *m*_try_ was defined as the square root of the number of variables, and the rest of the parameters were defined as the default value. Subsequently, OOB errors were calculated and the value of the best *n*_tree_ was obtained according to the lowest OOB error. Figure 8 shows that the minimum error and the standard error are the lowest, with 663 trees. Based on the optimal number of trees, *m*_try_ was re-selected by searching the values ranged from 50 to 75. The calculation results found that the *m*_try_ value should be defined as 61, because of the model had the lowest OOB classification error. Finally, a final classification model was established based on optimum *n*_tree_ and *m*_try_ values.

Table 1 shows that the accuracies for samples of calibration set were 96.77% for low latitude samples, 99.46% for mid-latitude samples, 94.62% for mid-high latitude samples, and 94.09% for high latitude samples. Additionally, the accuracies of samples of validation set were 91.49%, 95.74%, 94.68%, and 98.94% for four different latitudes samples, respectively.

**Figure 9:**
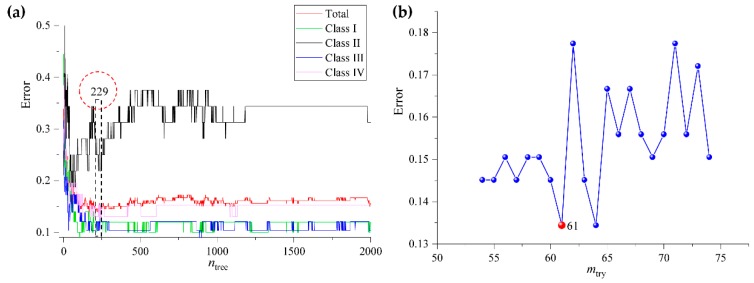
The *n*_tree_ (**a**) and *m*_try_ (**b**) screening of RF models based on stems fingerprints.

**Figure 10:**
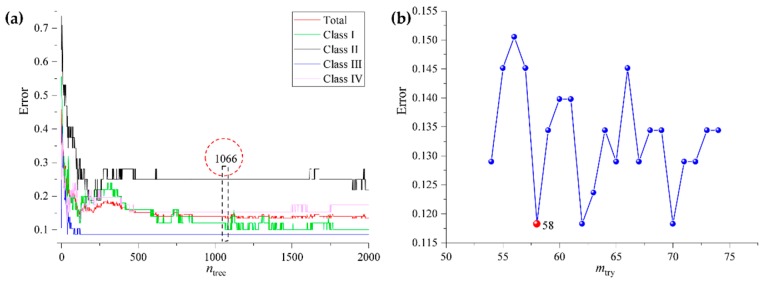
The *n*_tree_ (**a**) and *m*_try_ (**b**) screening of RF models based on leaves fingerprints.

Like previous investigations of the rhizome model, the data set of stems (280 samples and 4140 variables) and leaves (280 samples and 4140 variables) were separated into calibration sets and validation sets, respectively. Subsequently, RF calibration modes of stems (S_RF) and leaves (L_RF) were built. The optimum *n*_tree_ and *m*_try_ could be found in Figure 9 and Figure 10.

For the RF model of the stem, the accuracies of samples of calibration set of 92.47%, 94.62%, 93.01%, and 93.01% were achieved for low latitudes, mid-latitudes, mid-high latitudes, and high latitudes. Additionally, the accuracies of samples of validation set were 98.94%, 97.87%, 96.81%, and 97.87%, respectively (Table 2).

For RF model of the leaf, accuracies of 92.47%, 96.24%, 93.01%, and 94.62% were achieved for the calibration set. Additionally, accuracies of 85.11%, 93.62%, 89.36%, and 93.62% for the validation set (Table 3).

#### 2.2.2. OPLS-DA Classification

The OPLS-DA models of rhizomes (R_OPLS-DA), stems (S_OPLS-DA), and leaves (L_OPLS-DA) were constructed based on the same calibration and validation sets that were used in RF models. All of the models were constructed based on the internal seven-fold cross-validation and permutation plot could be found in Supplementary Materials.

Table S1 showed that the *R*^2^ of models ranged from 0.77 to 0.82 and the *Q*^2^ of models were larger than 0.50, which indicated that the OPLS-DA models were well fitted and better predictive. The permutation test results could be found in Figures S14–S16.

The classification results of R_OPLS-DA model showed (Table 4) accuracies of calibration set were 98.92% for all classes. Accuracies of validation set were 95.47%, 98.94%, 94.86%, and 97.87% for low latitudes, mid-latitudes, mid-high latitudes, and high latitudes samples, respectively. For S_OPLS-DA models (Table 4), although 98.92%, 99.46%, 98.92%, and 98.39% values of calibration set accuracies were obtained for samples that were grown in four different latitudes, a lower value of total accuracy rate of validation set was obtained (93.62%). Parameters of L_OPLS-DA model showed (Table 4) that the accuracies of the calibration set were 97.31%, 99.46%, 97.31%, and 98.39% for low latitude, mid-latitude, mid-high latitude, and high latitude samples, respectively. However, the total accuracy of the validation set was lower than the calibration set. Especially, for samples of class 1, the accuracy was only 88.30%.

Finally, we made a comprehensive comparison to the six models’ classification performance superiority on the basis of the above analysis. For the RF model, the order of calibration total accuracy was as follows: R_RF (96.24%) > L_RF (94.09%) > S_RF (93.28%). The order of validation total accuracy was as follows: S_RF (97.87%) > R_RF (95.21%) > L_RF (90.43%). For the OPL-DA model, the order of calibration total accuracy was as follows: R_OPL-DA (98.92%) and S_OPLS-DA (98.92%) > L_OPLS-DA (98.12%). The order of validation total accuracy was as follows: R_OPL-DA (96.81%) > S_OPLS-DA (93.62%) > L_OPLS-DA (92.55%). Classification models that were built by using leaf data set presented the worst performance from the accuracy point of view. Additionally, validation sets of the L_RF and L_OPL-DA model had lower Matthews correlation coefficient (MCC) values. By contrast, all of the models based on rhizome data set presented a better classification performance (total accuracy ranged from 95.21% to 98.92%). The best total accuracy occurred when rhizome data combined with the OPLS algorithm. We could find that phenomenon of imbalance category recognition in R_OPLS-DA model was better than other models from SE values, SP values, MCC values, and EFF value.

Although the classification performance for OPLS-DA and RF models on the basis of rhizome data set was good, the model classification ability, accuracy, sensitivity (SE), specificity (SP), MCC, and efficiency (EFF), need to be enhanced. In a further step, the feasibility of combining the information from rhizome, stem, and leaf fingerprint data for samples geographical traceability was investigated by low-level and mid-level data fusion strategies.

### 2.3. Geographic Authentication Based on Data Fusion Strategy

#### 2.3.1. Low-Level Data Fusion

**Figure 11:**
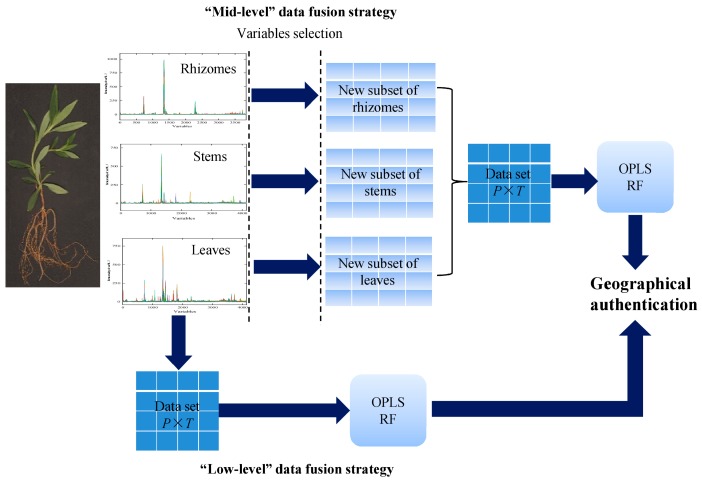
The workflow of geographical authentication of *G. rigescens* grown at different latitudes using data fusion strategy.

According to the method that was described in data preprocessing (Figure 11), fingerprint data sets of overground and underground organs as subsets were used to concatenate into a single data block (a new data set). In the case of the low-level strategy, four data sets, rhizome combined with stem (RS), rhizome combined with leaf (RL), stem combined with leaf (SL), and all data combined (RSL), were used to build RF (RS_RF, RL_RF, SL_RF, and RSL_RF) and OPLS-DA (RS_OPLS-DA, RL_OPLS-DA, SL_OPLS-DA, and RSL_OPLS-DA) models. For every data set, the samples were randomly selected as a calibration set and the rest of the samples were used as a validation set (finished by Kennard-Stone algorithm).

The optimum *n*_tree_ and *m*_try_ values were selected at first (Figure S8). Afterwards, final classification models were established based on the best values of arguments. From Table 5, it could be seen that the samples collected from four different latitudes were better discriminated by using RS data set and RSL data set. RS_RF model achieved 95.43% total accuracy for the calibration set and achieved 96.81% total accuracy for calibration set. RSL_RF model achieved 94.89% correctly for the calibration set and achieved 97.37% correctly for the calibration set. From a comparison with SE, SP, MCC, and EFF values of S_RF and L_RF models (Table 1 and Table 3), we found that the low-level data fusion strategy improved the phenomenon of imbalance category recognition in the RF model (Table 5). However, the total accuracy of models was not obviously improved.

The permutation plot of all models could be found in Supplementary Materials (Figures S17–S20). The classification results of OPLS-DA models based on low-level data fusion showed models’ *R*^2^ values ranged from 0.86 to 0.90 and *Q*^2^ values ranged from 0.74 to 0.80 (Table S2). Total accuracy rates of the calibration set of RS_OPLS-DA, RL_OPLS-DA, SL_OPLS-DA, and RSL_OPLS-DA were 99.46%, 99.73, 100.00%, and 99.73%, respectively (Table 6). Additionally, correct classification rates of validation sets varied from 97.34% to 98.40% (Table 6). The comparison parameters for SE, SP, MCC, and EFF (Table 4 and Table 6), the results highlight classification abilities of data fusion OPLS-DA models were better than the individual data set models. What is more, the RS_OPLS-DA model was the optimum classification model when using low-level data fusion strategy (Table 5 and Table 6).

#### 2.3.2. Mid-Level Data Fusion

At the end of the research, the feasibility for further optimizing the model parameters by feature subset selection and data fusion was investigated (Figure 11). Variables selection was one of the steps of the mid-level data fusion strategy. For the RF model, the “Boruta” algorithm was used to identify important chromatographic signal variables that significantly contributed to the classification performance. “Boruta” selection was finished based on three RM models that were built while using data sets of rhizomes (3839 variables), stems (4140 variables), and leaves (4140 variables), respectively. After comparing original attributes’ importance with importance achievable at random, 200 variables of rhizome data set, 305 variables of stem data set, and 359 of variables for leaf data set were retained as relevant features variables for sample discrimination (Figures S9–S11). Subsequently, those feature subsets were combined as a new data block and the fused data set (505 variables for RS, 559 variables for RL, 664 variables for SL, and 864 variables for RSL) was used to establish final classification models. The optimum *n*_tree_ and *m*_try_ values of RS_RF, RL_RF, SL_RF, and RSL_RF model could be found in Figure S12.

Table 7 lists the statistical results for the classification ability of the four RF models based on mid-level data fusion. The average accuracies of the calibration set and validation set were achieved for 96.44% and 97.21% by using RF algorithm. It is notable that the RL_RF model had accuracies that ranged from 94.09% to 99.46% in the calibration set and accuracy ranging from 96.81% to 100% in the validation set. In addition, parameters of SE (0.87–1.00), SP (0.94–1.00), MCC (0.87–1.00), and EFF (0.92–1.00) for each class of RL_RF model were higher than most RF classification models. As a result, mid-level data fusion strategy could eliminate the unnecessary variables, enhance model classification ability, and improve the phenomenon of imbalance category recognition in the RF model relative to low-level data fusion strategy.

For the OPLS-DA model, in front of all, three independent classification models were built while using original data sets of rhizome, stem, and leaf, respectively. Subsequently, the VIP value of variables in different classification models was calculated by SIMCA software. The results showed (Figure S12) that a total of 4486 variables (1309 variables selected from rhizome data set, 1538 variables selected from stem data set and 1639 variables selected from leaf data set) VIP values were greater than 1. Those variables with large importance for the geographical traceability of samples were combined into a new data set (2847 variables for RS, 2948 variables for RL, 3177 variables for SL, and 4486 variables for RSL) for final classification model building. The *R*^2^ and *Q*^2^ values and the permutation plot of RS_OPLS-DA, RL_OPLS-DA, SL_OPLS-DA, and RSL_OPLS-DA model were shown in Table S2 and Figures S21–S24.

The classification results showed that average accuracies of calibration and validation sets were achieved for 99.66% and 96.81%, respectively (Table 8). The four models exhibit good performances (MCC values ranged from 0.96 to 1.00 and EFF values ranged from 0.92 to 1.00 (Table 8). OPLS-DA models based on mid-level data fusion and low-level data fusion showed similar accuracy and model performance although feature selection was useful for reducing irrelevant variable when classifying samples.

Overall, it can be seen that there is an improvement in the results that were provided by data fusion when compared with performances of models based on independent data sets. When considering the similar accuracy and a higher SE, SP, MCC, and EFF values between calibration set and validation set, the RS_OPLS-DA models that were based on low-level data fusion strategy was the best performance.

## 3. Materials and Methods

### 3.1. Plant Material Collection

**Figure 12:**
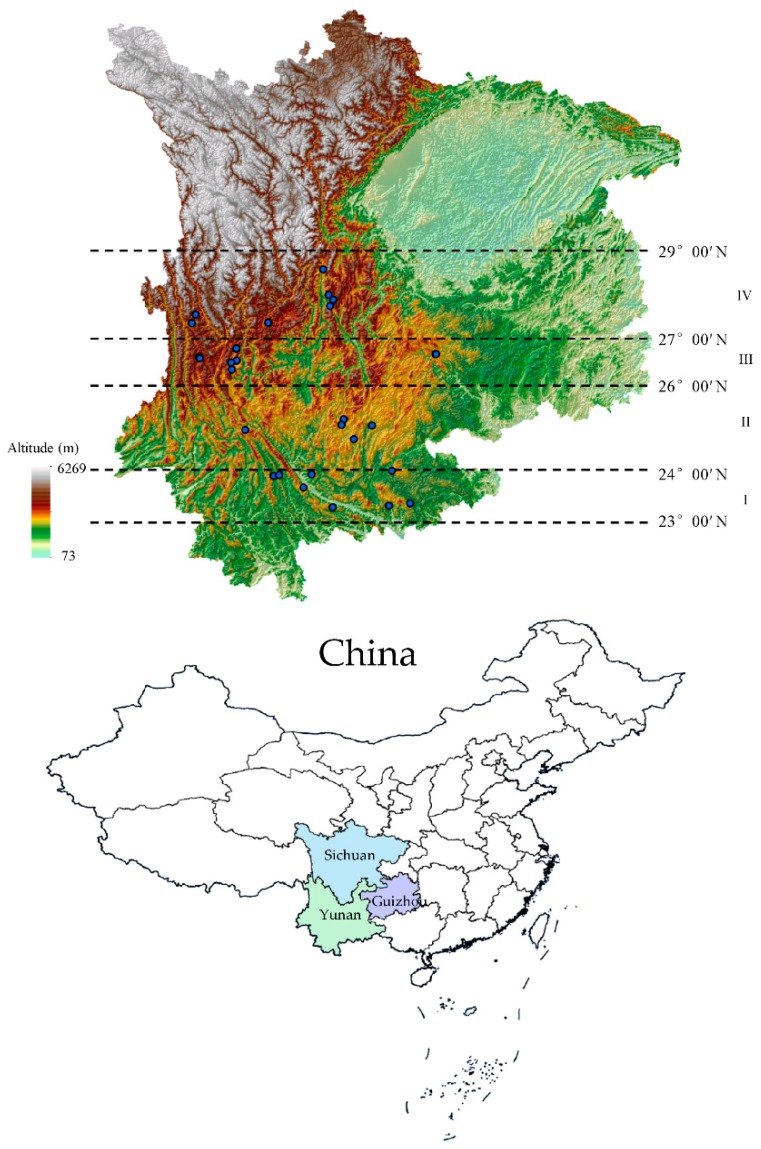
Geographical distribution of sample information.

Plant materials (29 population and 280 individuals) of *G. rigescens* were collected in the fall of 2012 and 2013 at the time of local traditional harvest period, at the different location of Yunnan, Guizhou, and Sichuan (Figure 12). Four producing areas were divided according to the location of population. (I) low latitudes area, with latitudes ranging from 23.92–23.66° N, South of Yunnan (eight population and 76 individuals), (II) mid-latitude area, with latitudes ranges from 24.95–25.06° N, Middle of Yunnan (five population and 48 individuals), (III) mid-high latitude area, with latitudes ranges from 26.49–26.64° N, Northwest of Yunnan and West of Guizhou (nine population and 76 individuals 87), and (IV) high latitude area, with latitudes ranges from 27.34–28.52° N, Hengduan Mountains Region of Yunnan and mountainous regions of Southwest of Sichuan (seven population and 69 individuals). The fresh materials were authenticated and transported to the laboratory of Yuxi normal University. Subsequently, samples were wash cleaning and dried at 50 °C as soon as possible. At last, all samples (rhizomes, stems and leaves) were stored in a relatively dry environment prior to the extraction procedure.

### 3.2. Chemicals and Reagents

HPLC-grade acetonitrile, methanol (MeOH) were supplied by Thermo Fisher Scientific (Waltham, MA, USA). HPLC-grade formic acid was purchased from Sigma-Aldrich (Steinheim, Germany). Deionized water was obtained from Wahaha Group Co., Ltd. (Hangzhou, Zhejiang, China). The primary grade reference standards loganin (purity: ≥98%), 6′-*O*-β-d-glucopyranosylgentiopicroside (purity: ≥98%), swertiamarine (purity: ≥98%), gentiopicroside (purity: ≥98%), and sweroside (purity: ≥98%) were purchased from the Chinese National Institute for Food and Drug Control (Beijing, China), Shanghai Shifeng Biological Technology (Shanghai, China), respectively.

### 3.3. Sample Preparation

The dried samples (rhizomes, stems, and leaves) were ground and then passed through a 100 mesh sieves. Each sample powder (25 mg) was accurately weighed and extracted while using 1.5 mL 80% methanol-water solution, at 25 °C. The samples were extracted while using an Ultrasonic extractor for 40 min. The final extract was filtered with a 0.22 μm syringe filter into an HPLC vial and then subjected to HPLC analysis [16,58].

### 3.4. Instrumentation and HPLC Analysis

Chromatographic analyses were performed with an Agilent 1260 Infinity LC system (Agilent Technologies, Santa Clara, CA, USA), which was equipped with a G1315D diode-array detector, a G1329B ALS autosampler, and a thermostated column compartment. The HPLC fingerprint was recorded by Chemstation software (Agilent Technologies, Waldbron, Germany).

The analytical separation was adopted from a published method for chemical fingerprinting analysis [16]. The separation was achieved on a reversed phase C18 (Agilent Intersil, 5 µm, 4.6 × 150 mm) column (Agilent, Santa Clara, CA, USA). The composition of the mobile phase was: (A) 0.1% phosphoric acid in water and (B) 100% acetonitrile. The separation was as follows: 0.00–2.50 min: 7–10% B, 2.50–20.00 min: 10–26% B, 20.00–29.02 min: 26–58.3% B, 29.02–30.00 min: 58.3–90% B. The column was subsequently washed with 90% B and re-equilibrated with 7% B prior to injection of the next sample. The flow rate was 1.0 mL/min and the column temperature was 30 °C. The injection volume was 5 µL and the detective wavelength of UV spectra was set at 241 nm. Chromatographic data was processed while using OpenLab software (Agilent Technologies) [16,58].

### 3.5. Data Analysis

HPLC fingerprints from the 280 rhizome samples, 280 stem samples, and 280 leaf samples, a total of 840 fingerprint data was exported in CSV format and imported to MATLAB R2018b (The MathWorks, Inc., Natick, MA, USA), which was used for correlation optimized warping (COW) alignment preprocessing of chromatographic fingerprint. MATLAB code of COW is freely available from www.models.kvl.dk. The preprocessing fingerprint was analyzed in the following work [59].

Exploratory data analysis (EDA) is necessary for building predictive models [60,61]. It can help in determining interesting correlations among all of the samples or variables and summarize data sets main characteristics [60]. Principal component analysis (PCA) is a popular primary tool in EDA [61,62]. It is often used to visualize the relatedness between samples and explains the variance in the data. Hence, PCA, as an unsupervised pattern recognition technique, was widely used to extract key information from chemical fingerprint for geographical origin or Modelling Research [61].

Unlike PCA, orthogonal partial least squares discriminant analysis (OPLS-DA) is a supervised pattern recognition technique. As an extension of PLS, an inbuilt orthogonal signal correction filter was incorporated in the OPLS-DA model [56]. This algorithm effectively divides the X variable into two parts: one part that is related to class information (Y-predictive) and the other is orthogonal or unrelated to class information (Y-uncorrelated). Therefore, interpretability and prediction performance of the model was enhanced [56].

Random forest (RF) is another supervised pattern recognition technique utilized in the study. RF is an ensemble learning method [55]. A large number of trees were produced by RF algorithm in order to improve model predictive ability, and trees’ decision results were combined as final decision results. In other words, the more trees built in the random forest classifier, the higher accuracy could be achieved. However, many researches showed that an optimum tree number was of great importance in modeling classification performance [33,46].

In this work, exploratory data analysis of HPLC fingerprints of *G. rigescens* grown in four different latitudes was finished with PCA. Two supervised pattern recognition techniques, OPLS-DA and RF, were applied to build classification models for *G. rigescens* producing areas. SIMCA 14.1 software managed PCA and OPLS-DA (Umetrics AB, Umea, Sweden). RF classification models were established with R 3.5.1 program and package randomForest (Version 4.6-14) [63].

#### Data Fusion Strategy

In the case of low-level fusion strategy (Figure 11), different subsets HPLC fingerprint data matrix of rhizomes, stems, and leaves) are straightforwardly concatenated and compiled into a new chromatographic data matrix for subsequent classification model construction [45,46]. Furthermore, each subset must be totally aligned and keep all the variables on the same scale before subsets reconnection [45,46].

In the case of mid-level fusion (Figure 11), the first step of data treatment is feature selection that is based on rhizomes, stems, or leaves classification models. When compared with the raw data sets, feature selection of subsets minimizes the data content and reduces data dimensions. Subsequently, new subsets of rhizomes, stems, and leaves were rebuilt while using variables of feature selection [45]. At last, those subsets are concatenated and compiled into a final data matrix for model construction [45].

In the research, relevant variables of RF classification models were determined by the R software package Boruta [64], and VIP was used for important variables selection of OPLS-DA [65].

### 3.6. Model Evaluation

Five parameters, including accuracy (ACC), sensitivity (SE), specificity (SP), efficiency (EFF), and Matthews correlation coefficient (MCC) were applied to evaluate the identification ability of RF and OPLS-DA models. The ruggedness of OPLS-DA model was investigated through 200 times permutation tests. Furthermore, cumulative prediction ability (Q^2^), cumulative interpretation ability (R^2^), root mean square error of estimation (RMSEE), root mean square error of cross-validation (RMSECV), and root mean square error of prediction (RMSEP) were important evaluation indexes for the predictive power of OPLS-DA model [33,66].

Values of TP (Correctly identified samples of positive class), TN (correctly identified samples of negative class), FN (incorrectly identified samples of positive class), and FP (incorrectly identified samples of negative class) were calculated according to confusion matrixes of classification models. Subsequently, ACC, SE, SP, EFF, and MCC were calculated while using Equations (1)–(5) and values of Q^2^, R^2^, RMSEE, RMSECV, and RMSEP computed by software SIMCA 14.1.

(1) $$\mathrm{ACC}=\frac{\left(\mathrm{TN}+\mathrm{TP}\right)}{\left(\mathrm{TP}+\mathrm{TN}+\mathrm{FP}+\mathrm{FN}\right)}$$

(2) $$\mathrm{SE}=\frac{\mathrm{TP}}{\left(\mathrm{TP}+\mathrm{FN}\right)}$$

(3) $$\mathrm{SP}=\frac{\mathrm{TN}}{\left(\mathrm{TN}+\mathrm{FP}\right)}$$

(4) $$\mathrm{EFF}=\sqrt{\mathrm{SE}\times \mathrm{SP}}$$

(5) $$\mathrm{MCC}=\frac{\left(\mathrm{TP}\times \mathrm{TN}-\mathrm{FP}\times \mathrm{FN}\right)}{\sqrt{\left(\mathrm{TP}+\mathrm{FP}\right)\left(\mathrm{TP}+\mathrm{FN}\right)\left(\mathrm{TN}+\mathrm{FP}\right)\left(\mathrm{TN}+\mathrm{FN}\right)}}$$

For model performance, lower values of RMSEE, RMSECV, and RMSEP mean better predictive ability for the models. Conversely, the closer that values of ACC, SE, SP, EFF, MCC, and Q^2^, R^2^ are to 1, the more well performance the model is.

## 4. Conclusions

The findings in this study showed that *G. rigescens* chemical profiles were influenced by the latitude gradients of producing areas and lower latitudes and higher latitudes samples seemed to be clearly distinguishable. According to the score plots of PCA and OPLS-DA, the phytochemical geographic variation of the overground and underground part along the latitude gradients was visualized. Subsequently, the potential of fingerprint data obtained while using HPLC-DAD to discriminate and classify *G. rigescens* grown in four different latitudes was investigated. Additionally, RF and OPLS-DA models were used to develop an effective way for geographical traceability of the *G. rigescens* that were grown in four different latitudes. When using independent data sets to build models, rhizomes data set combined with OPLS-DA presented the best performance with a classification accuracy of calibration and validation set varied from 94.68% to 98.94%. In a further step, the feasibility of combining the chromatographic fingerprint data from overground and underground organs was investigated based on two kinds of data fusion strategies in order to improve the performance of classification models: low-level and mid-level. Notably, classification performances of OPLS-DA models were efficiently improved by low-level data fusion strategy and better performances of RF models appeared to be achieved by mid-level data fusion strategy. Although satisfactory results were obtained with both RF and OPLS-DA based on two kinds of data fusion strategies, OPLS-DA combined with rhizome-stem fusion data set was the optimum model for discriminating *G. rigescens* samples according to their grown latitudes, with an accuracy of (97.87–100.00%), SE of (0.96–1.00), SP of (0.98–1.00), MCC of (0.95–1.00), and EFF of (0.97–1.00).

## Figures and Tables

**Table 1:** The major parameters of random forest (RF) model based on rhizomes data set.

Model	Performance	Calibration Set				Validation Set			
		I	II	III	IV	I	II	III	IV
R_RF	ACC (%)	96.77	99.46	94.62	94.09	91.49	95.74	94.68	98.94
	SE	0.92	0.97	0.93	0.89	0.92	0.75	0.93	0.96
	SP	0.99	1.00	0.95	0.96	0.91	1.00	0.95	1.00
	MCC	0.92	0.98	0.88	0.84	0.80	0.84	0.88	0.97
	EFF	0.95	0.98	0.94	0.92	0.92	0.87	0.94	0.98

**Table 2:** The major parameters of RF model based on stems data set.

Model	Performance	Calibration Set				Validation Set			
		I	II	III	IV	I	II	III	IV
S_RF	ACC (%)	92.47	94.62	93.01	93.01	98.94	97.87	96.81	97.87
	SE	0.92	0.69	0.91	0.87	1.00	0.88	1.00	0.91
	SP	0.93	1.00	0.94	0.95	0.99	1.00	0.95	1.00
	MCC	0.82	0.80	0.84	0.81	0.97	0.92	0.93	0.94
	EFF	0.92	0.83	0.93	0.91	0.99	0.94	0.98	0.96

**Table 3:** The major parameters of RF model based on leaves data set.

Model	Performance	Calibration Set				Validation Set			
		I	II	III	IV	I	II	III	IV
L_RF	ACC (%)	92.47	96.24	93.01	94.62	85.11	93.62	89.36	93.62
	SE	0.94	0.78	0.91	0.85	0.88	0.69	0.86	0.74
	SP	0.92	1.00	0.94	0.98	0.84	0.99	0.91	1.00
	MCC	0.82	0.86	0.84	0.85	0.67	0.76	0.76	0.83
	EFF	0.93	0.88	0.93	0.91	0.86	0.82	0.88	0.86

**Table 4:** The major parameters of OPLS-DA models.

Model	Performance	Calibration Set				Validation Set			
		I	II	III	IV	I	II	III	IV
R_OPLS-DA	ACC (%)	98.92	98.92	98.92	98.92	95.74	98.94	94.68	97.87
	SE	0.98	0.97	0.98	0.98	0.92	0.94	0.93	0.96
	SP	0.99	0.99	0.99	0.99	0.97	1.00	0.95	0.99
	MCC	0.97	0.96	0.97	0.97	0.89	0.96	0.88	0.94
	EFF	0.99	0.98	0.99	0.99	0.95	0.97	0.94	0.97
S_OPLS-DA	ACC (%)	98.92	99.46	98.92	98.39	91.49	93.62	91.49	97.87
	SE	1.00	0.97	1.00	0.93	0.92	0.81	0.83	0.91
	SP	0.99	1.00	0.98	1.00	0.91	0.96	0.95	1.00
	MCC	0.97	0.98	0.98	0.96	0.80	0.77	0.80	0.94
	EFF	0.99	0.98	0.99	0.97	0.92	0.88	0.89	0.96
L_OPLS-DA	ACC (%)	97.31	99.46	97.31	98.39	88.30	95.74	92.55	93.62
	SE	0.94	1.00	0.93	1.00	0.81	0.88	0.90	0.83
	SP	0.99	0.99	0.99	0.98	0.91	0.97	0.94	0.97
	MCC	0.93	0.98	0.94	0.96	0.71	0.85	0.83	0.82
	EFF	0.96	1.00	0.96	0.99	0.86	0.92	0.92	0.90

**Table 5:** The major parameters of RF models based on low-level data fusion strategy.

Model	Class	Calibration Set				Validation Set			
		I	II	III	IV	I	II	III	IV
RS_RF	ACC (%)	96.77	98.92	92.47	93.55	95.74	97.87	95.74	97.87
	SE	0.92	0.94	0.91	0.87	0.92	0.88	0.97	0.96
	SP	0.99	1.00	0.93	0.96	0.97	1.00	0.95	0.99
	MCC	0.92	0.96	0.83	0.83	0.89	0.92	0.90	0.94
	EFF	0.95	0.97	0.92	0.91	0.95	0.94	0.96	0.97
RL_RF	ACC (%)	94.09	98.39	93.01	96.24	87.23	94.68	91.49	92.55
	SE	0.90	0.91	0.91	0.91	0.96	0.75	0.86	0.70
	SP	0.96	1.00	0.94	0.98	0.84	0.99	0.94	1.00
	MCC	0.85	0.94	0.84	0.90	0.74	0.80	0.80	0.80
	EFF	0.93	0.95	0.93	0.95	0.90	0.86	0.90	0.83
SL_RF	ACC (%)	93.55	95.70	92.47	93.55	90.43	96.81	96.81	94.68
	SE	0.94	0.75	0.91	0.85	0.92	0.88	0.93	0.83
	SP	0.93	1.00	0.93	0.96	0.90	0.99	0.98	0.99
	MCC	0.84	0.84	0.83	0.82	0.78	0.88	0.92	0.85
	EFF	0.94	0.87	0.92	0.90	0.91	0.93	0.96	0.90
RSL_RF	ACC (%)	95.70	99.46	91.94	92.47	94.68	96.81	100.00	97.87
	SE	0.94	0.97	0.86	0.85	0.86	1.00	1.00	0.96
	SP	0.96	1.00	0.95	0.95	0.98	0.96	1.00	0.99
	MCC	0.89	0.98	0.81	0.80	0.87	0.88	1.00	0.94
	EFF	0.95	0.98	0.90	0.90	0.92	0.98	1.00	0.97

**Table 6:** The major parameters of OPLS-DA models based on low-level data fusion strategy.

Model	Class	Calibration Set				Validation Set			
		I	II	III	IV	I	II	III	IV
RS_OPLS-DA	ACC (%)	99.46	100.00	99.46	98.92	97.87	98.94	97.87	98.94
	SE	1.00	1.00	1.00	0.96	0.96	1.00	0.97	0.96
	SP	0.99	1.00	0.99	1.00	0.99	0.99	0.98	1.00
	MCC	0.99	1.00	0.99	0.97	0.95	0.96	0.95	0.97
	EFF	1.00	1.00	1.00	0.98	0.97	0.99	0.98	0.98
RL_OPLS-DA	ACC (%)	99.46	100.00	100.00	99.46	95.74	97.87	97.87	97.87
	SE	1.00	1.00	1.00	0.98	0.88	1.00	0.97	0.96
	SP	0.99	1.00	1.00	1.00	0.99	0.97	0.98	0.99
	MCC	0.99	1.00	1.00	0.99	0.89	0.93	0.95	0.94
	EFF	1.00	1.00	1.00	0.99	0.93	0.99	0.98	0.97
SL_OPLS-DA	ACC (%)	100.00	100.00	100.00	100.00	94.68	98.94	97.87	97.87
	SE	1.00	1.00	1.00	1.00	1.00	0.94	0.93	0.91
	SP	1.00	1.00	1.00	1.00	0.93	1.00	1.00	1.00
	MCC	1.00	1.00	1.00	1.00	0.88	0.96	0.95	0.94
	EFF	1.00	1.00	1.00	1.00	0.96	0.97	0.96	0.96
RSL_OPLS-DA	ACC (%)	99.46	100.00	100.00	99.46	96.81	98.94	97.87	97.87
	SE	1.00	1.00	1.00	0.98	0.92	1.00	0.97	0.96
	SP	0.99	1.00	1.00	1.00	0.99	0.99	0.98	0.99
	MCC	0.99	1.00	1.00	0.99	0.92	0.96	0.95	0.94
	EFF	1.00	1.00	1.00	0.99	0.95	0.99	0.98	0.97

**Table 7:** The major parameters of RF models based on mid-level data fusion strategy.

Model	Class	Calibration Set				Validation Set			
		I	II	III	IV	I	II	III	IV
RS_RF	ACC (%)	99.46	99.46	94.09	95.16	98.94	100.00	96.81	97.87
	SE	0.98	0.97	0.95	0.87	1.00	1.00	0.97	0.91
	SP	1.00	1.00	0.94	0.98	0.99	1.00	0.97	1.00
	MCC	0.99	0.98	0.87	0.87	0.97	1.00	0.93	0.94
	EFF	0.99	0.98	0.94	0.92	0.99	1.00	0.97	0.96
RL_RF	ACC (%)	95.70	96.77	96.24	97.31	91.49	98.94	91.49	94.68
	SE	0.92	0.88	0.97	0.93	0.92	1.00	0.86	0.78
	SP	0.97	0.99	0.96	0.99	0.91	0.99	0.94	1.00
	MCC	0.89	0.88	0.91	0.93	0.80	0.96	0.80	0.86
	EFF	0.94	0.93	0.96	0.96	0.92	0.99	0.90	0.88
SL_RF	ACC (%)	95.16	96.77	93.55	96.24	97.87	100.00	98.94	96.81
	SE	0.94	0.81	0.95	0.89	0.96	1.00	1.00	0.91
	SP	0.96	1.00	0.93	0.99	0.99	1.00	0.98	0.99
	MCC	0.88	0.88	0.86	0.90	0.95	1.00	0.98	0.91
	EFF	0.95	0.90	0.94	0.94	0.97	1.00	0.99	0.95
RSL_RF	ACC (%)	97.85	99.46	94.09	95.70	96.81	100.00	95.74	98.94
	SE	0.94	0.97	0.93	0.91	0.96	1.00	0.93	0.96
	SP	0.99	1.00	0.95	0.97	0.97	1.00	0.97	1.00
	MCC	0.94	0.98	0.86	0.88	0.92	1.00	0.90	0.97
	EFF	0.97	0.98	0.94	0.94	0.97	1.00	0.95	0.98

**Table 8:** The major parameters of OPLS-DA models based on mid-level data fusion strategy.

Model	Class	Calibration Set				Validation Set			
		I	II	III	IV	I	II	III	IV
RS_OPLS-DA	ACC (%)	100.00	100.00	99.46	99.46	93.62	97.87	94.68	98.94
	SE	1.00	1.00	1.00	0.98	0.88	1.00	0.90	0.96
	SP	1.00	1.00	0.99	1.00	0.96	0.97	0.97	1.00
	MCC	1.00	1.00	0.99	0.99	0.84	0.93	0.87	0.97
	EFF	1.00	1.00	1.00	0.99	0.92	0.99	0.93	0.98
RL_OPLS-DA	ACC (%)	100.00	100.00	99.46	99.46	96.81	97.87	97.87	98.94
	SE	1.00	1.00	1.00	0.98	0.92	1.00	0.97	0.96
	SP	1.00	1.00	0.99	1.00	0.99	0.97	0.98	1.00
	MCC	1.00	1.00	0.99	0.99	0.92	0.93	0.95	0.97
	EFF	1.00	1.00	1.00	0.99	0.95	0.99	0.98	0.98
SL_OPLS-DA	ACC (%)	100.00	100.00	98.92	98.92	93.62	97.87	94.68	96.81
	SE	1.00	1.00	0.98	0.98	0.92	0.94	0.90	0.91
	SP	1.00	1.00	0.99	0.99	0.94	0.99	0.97	0.99
	MCC	1.00	1.00	0.97	0.97	0.85	0.92	0.87	0.91
	EFF	1.00	1.00	0.99	0.99	0.93	0.96	0.93	0.95
RSL_OPLS-DA	ACC (%)	100.00	100.00	99.46	99.46	95.74	98.94	96.81	97.87
	SE	1.00	1.00	1.00	0.98	0.92	1.00	0.93	0.96
	SP	1.00	1.00	0.99	1.00	0.97	0.99	0.98	0.99
	MCC	1.00	1.00	0.99	0.99	0.89	0.96	0.92	0.94
	EFF	1.00	1.00	1.00	0.99	0.95	0.99	0.96	0.97

## Supplementary Materials

The following are available online. Figure S1: Variation of stems score plots along the latitude gradients, Figure S2: Variation of stems score plots between the adjacent latitudes, Figure S3: Variation of leaves score plots along the latitude gradients, Figure S4: Variation of leaves score plots between the adjacent latitudes, Figure S5: Permutation plot of the OPLS-DA of rhizome samples, Figure S6: Permutation plot of the OPLS-DA of stem samples, Figure S7: Permutation plot of the OPLS-DA of leaf samples, Figure S8: The *n*_tree_ and *m*_try_ screening of RF models based on low-level data fusion strategy, Figure S9: Result of variables selection of rhizome fingerprint data based on “Boruta” algorithm, Figure S10. Result of variables selection of stem fingerprint data based on “Boruta” algorithm, Figure S11: Result of variables selection of leaf fingerprint data based on “Boruta” algorithm, Figure S12: The *n*_tree_ and *m*_try_ screening of RF models based on mid-level data fusion strategy, Figure S13: The importance variables of OPLS-DA models of rhizomes, stems and leaves fingerprints data, Figure S14: Permutation testing (200 times) of the R_OPLS-DA model, Figure S15: Permutation testing (200 times) of the S_OPLS-DA model, Figure S16: Permutation testing (200 times) of the L_OPLS-DA model, Figure S17: Permutation testing (200 times) of the RS_OPLS-DA model based on low-level data fusion, Figure S18: Permutation testing (200 times) of the RL_OPLS-DA model based on low-level data fusion, Figure S19: Permutation testing (200 times) of the SL_OPLS-DA model based on low-level data fusion, Figure S20: Permutation testing (200 times) of the RSL_OPLS-DA model based on low-level data fusion, Figure S21: Permutation testing (200 times) of the RS_OPLS-DA model based on mid-level data fusion, Figure S22: Permutation testing (200 times) of the RL_OPLS-DA model based on mid-level data fusion, Figure S23: Permutation testing (200 times) of the SL_OPLS-DA model based on mid-level data fusion, Figure S24: Permutation testing (200 times) of the RSL_OPLS-DA model based on mid-level data fusion, Table S1: The evaluation indexes for predictive power of OPLS-DA model of rhizome, stem and leaf, Table S2: The evaluation indexes for predictive power of OPLS-DA models based on low-level and mid-level data fusion strategies.
