# Supplementary material for: Assessing Geographical Origin of Gentiana Rigescens Using Untargeted Chromatographic Fingerprint, Data Fusion and Chemometrics
Source: Molecules. 2019 Jul 14;24(14):2562. doi: 10.3390/molecules24142562 (PMC6680800; doi:10.3390/molecules24142562)
Supplement: Supplementary file 1 [file molecules-24-02562-s001.pdf]

# Assessing geographical origin of *Gentiana rigescens* using untargeted chromatographic fingerprint, data fusion and chemometrics

**Tao Shen [1, 2, 3], Hong Yu [1, 2]*, Yuan-Zhong Wang [4]**

[1] Yunnan Herbal Laboratory, Institute of Herb Biotic Resources, School of Life and Sciences, Yunnan University, Kunming 650091, China; st_yxnu@126.com
[2] The International Joint Research Center for Sustainable Utilization of Cordyceps Bioresouces in China and Southeast Asia, Yunnan University, Kunming 650091, China
[3] College of Chemistry, Biological and Environment, Yuxi Normal University,Yu'xi 653100, Yunnan, China
[4] College of Traditional Chinese Medicine, Yunnan University of Chinese Medicine, Kunming 650500, China
* Correspondence: hongyu@ynu.edu.cn, herbfish@163.com (H.Y.); Tel:+86-0871-68182671

**Figure captions**

**Figure S1.** Variation of stems score plots along the latitude gradients

**Figure S2.** Variation of stems score plots between the adjacent latitudes

**Figure S3.** Variation of leaves score plots along the latitude gradients

**Figure S4.** Variation of leaves score plots between the adjacent latitudes

**Figure S5.** Permutation plot of the OPLS-DA of rhizome samples

**Figure S6.** Permutation plot of the OPLS-DA of stem samples

**Figure S7.** Permutation plot of the OPLS-DA of leaf samples

**Figure S8.** The $n_{tree}$ and $m_{try}$ screening of RF models based on low-level data fusion strategy

**Figure S9.** Result of variables selection of rhizome fingerprint data based on "Boruta" algorithm (red triangle = relevant features variables)

**Figure S10.** Result of variables selection of stem fingerprint data based on "Boruta" algorithm

**Figure S11.** Result of variables selection of leaf fingerprint data based on "Boruta" algorithm

**Figure S12.** The $n_{tre}$ and $m_{try}$ screening of RF models based on mid-level data fusion strategy

**Figure S13.** The importance variables of OPLS-DA models of rhizomes, stems and leaves fingerprints data

**Figure S14.** Permutation testing (200 times) of the R_OPLS-DA model

**Figure S15.** Permutation testing (200 times) of the S_OPLS-DA model

**Figure S16.** Permutation testing (200 times) of the L_OPLS-DA model

**Figure S17.** Permutation testing (200 times) of the RS_OPLS-DA model based on low-level data fusion

**Figure S18.** Permutation testing (200 times) of the RL_OPLS-DA model based on low-level data fusion

**Figure S19.** Permutation testing (200 times) of the SL_OPLS-DA model based on low-level data fusion

**Figure S20.** Permutation testing (200 times) of the RSL_OPLS-DA model based on low-level data fusion

**Figure S21.** Permutation testing (200 times) of the RS_OPLS-DA model based on mid-level data fusion

**Figure S22.** Permutation testing (200 times) of the RL_OPLS-DA model based on mid-level data fusion

**Figure S23.** Permutation testing (200 times) of the SL_OPLS-DA model based on mid-level data fusion

**Figure S24.** Permutation testing (200 times) of the RSL_OPLS-DA model based on mid-level data fusion

## Table captions

**Table S1.** The evaluation indexes for predictive power of OPLS-DA model of rhizome, stem and leaf

**Table S2.** The evaluation indexes for predictive power of OPLS-DA models based on low-level and mid-level data fusion strategies

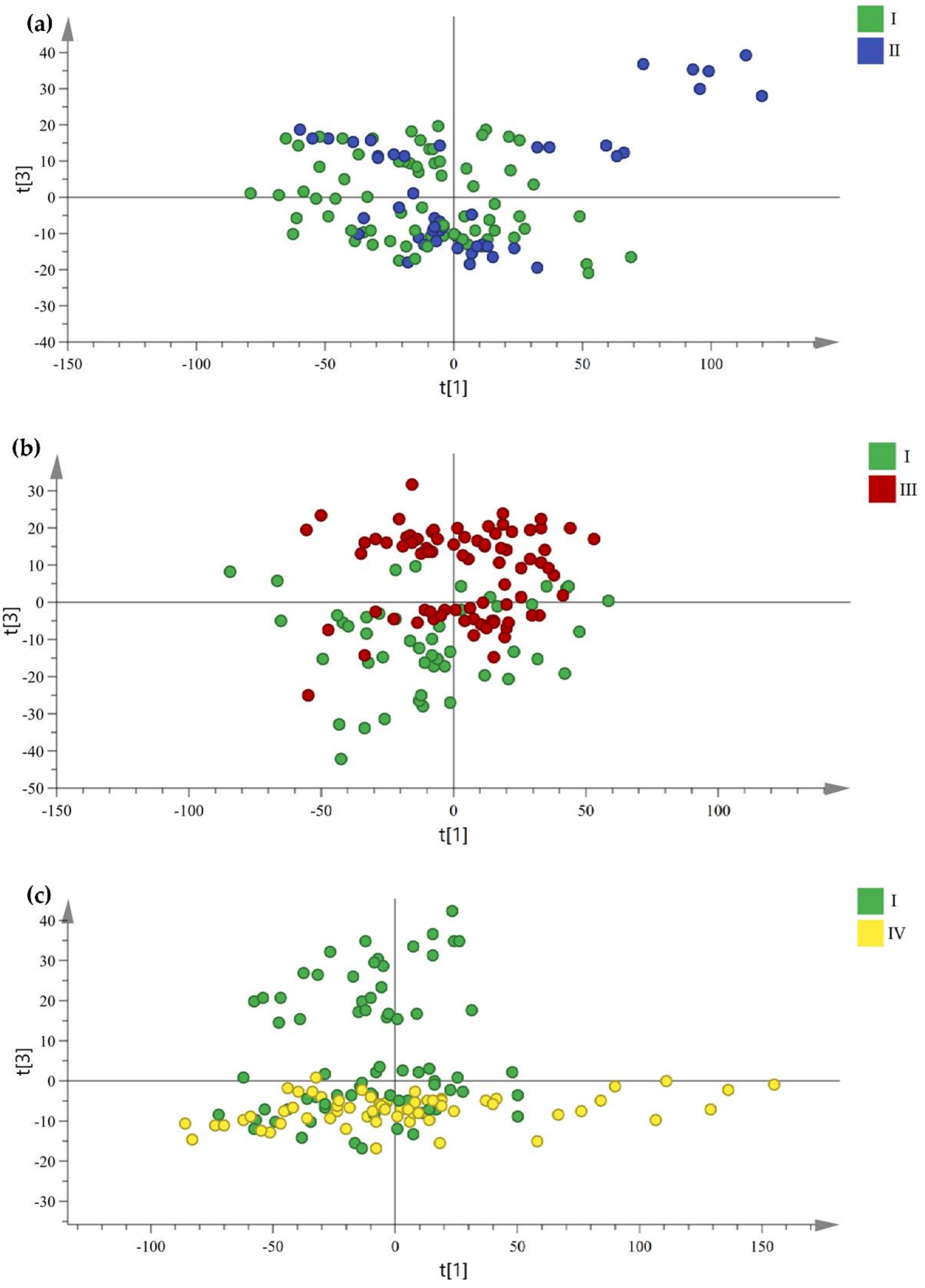


**Figure S1.** Variation of stems score plots along the latitude gradients (green circles = low latitudes area, 23.92-23.66 °N, blue circles = mid latitude area, 24.95-25.06 °N, red circles = mid-high latitude area, 26.49-26.64 °N, yellow circles = high latitude area, 27.34-28.52 °N)

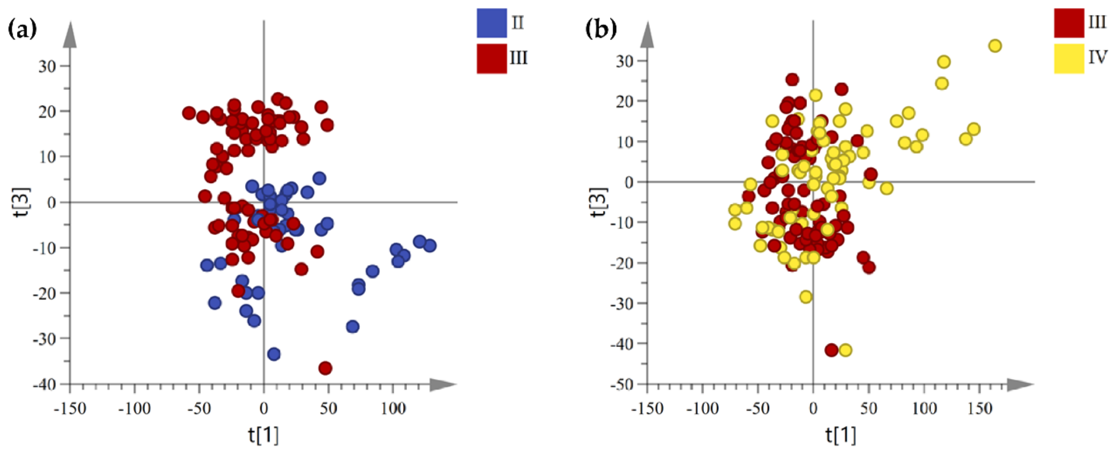


**Figure S2.** Variation of stems score plots between the adjacent latitudes (blue circles = mid latitude area, 24.95-25.06 °N, red circles = mid-high latitude area, 26.49-26.64 °N, yellow circles = high latitude area, 27.34-28.52 °N)

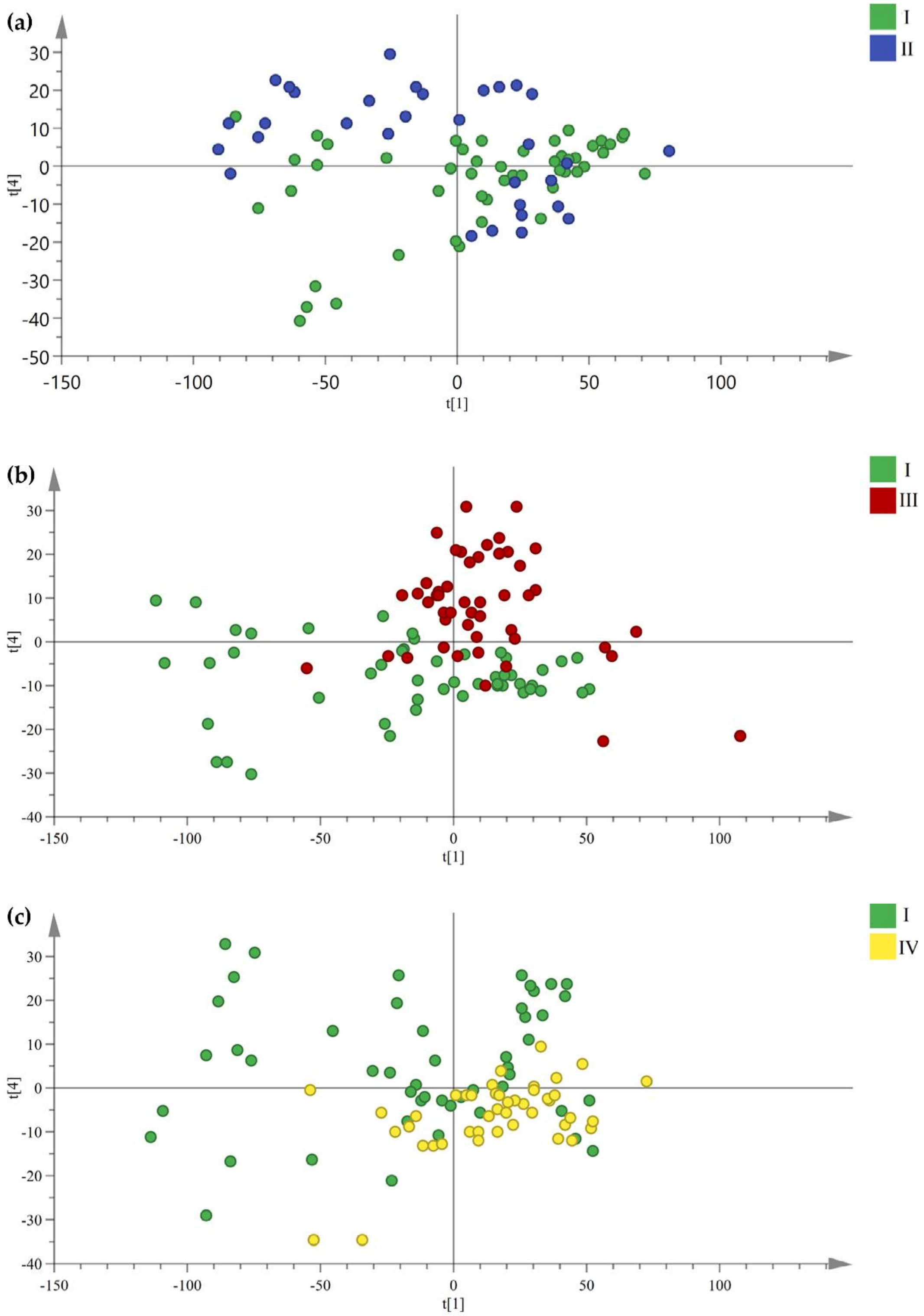


**Figure S3.** Variation of leaves score plots along the latitude gradients (green circles = low latitudes area, 23.92-23.66 °N, blue circles = mid latitude area, 24.95-25.06 °N, red circles = mid-high latitude area, 26.49-26.64 °N, yellow circles = high latitude area, 27.34-28.52 °N)

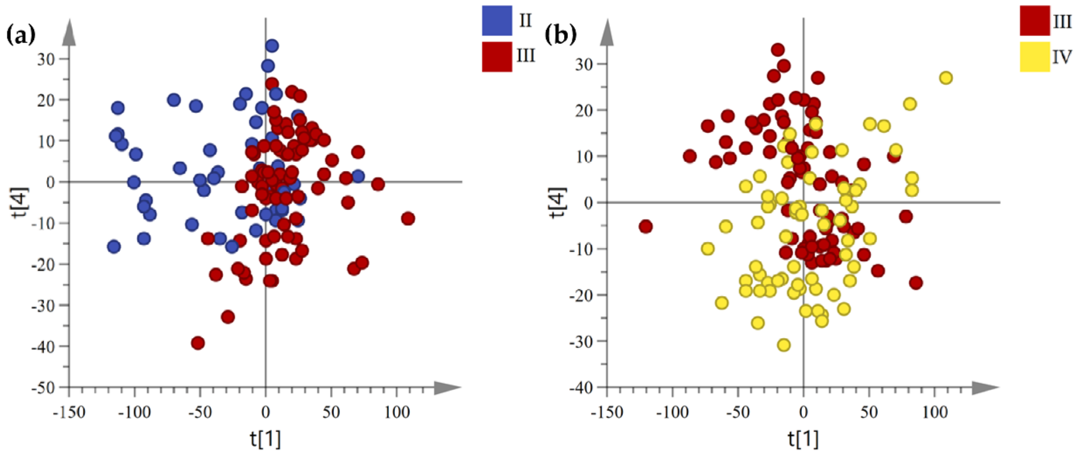


**Figure S4.** Variation of leaves score plots between the adjacent latitudes (blue circles = mid-latitude area, 24.95-25.06 °N, red circles = mid-high latitude area, 26.49-26.64 °N, yellow circles = high latitude area, 27.34-28.52 °N)

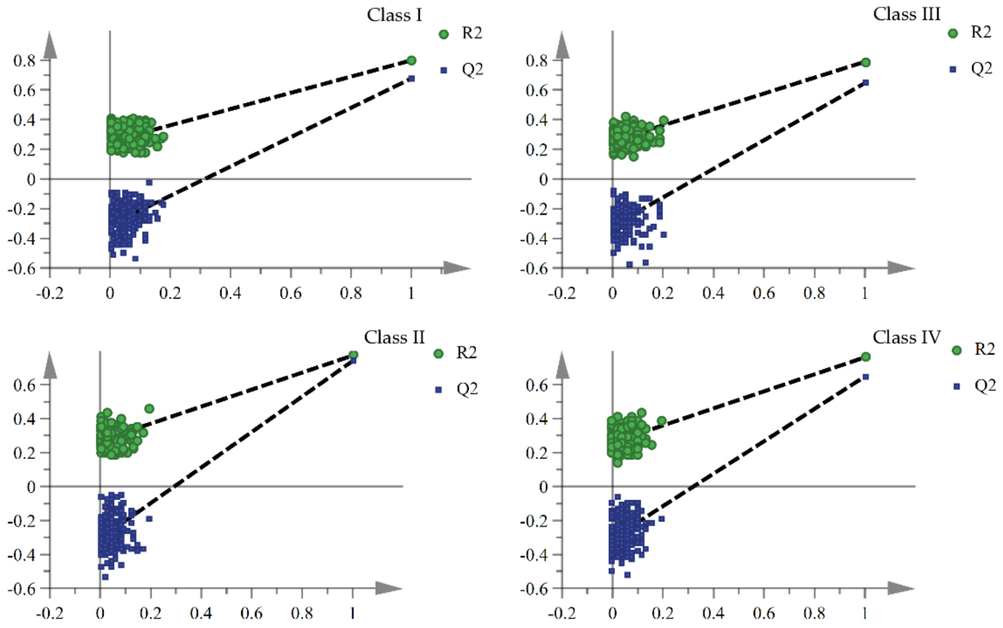


**Figure S5.** Permutation plot of the OPLS-DA of rhizome samples (Number of permutations = 200)

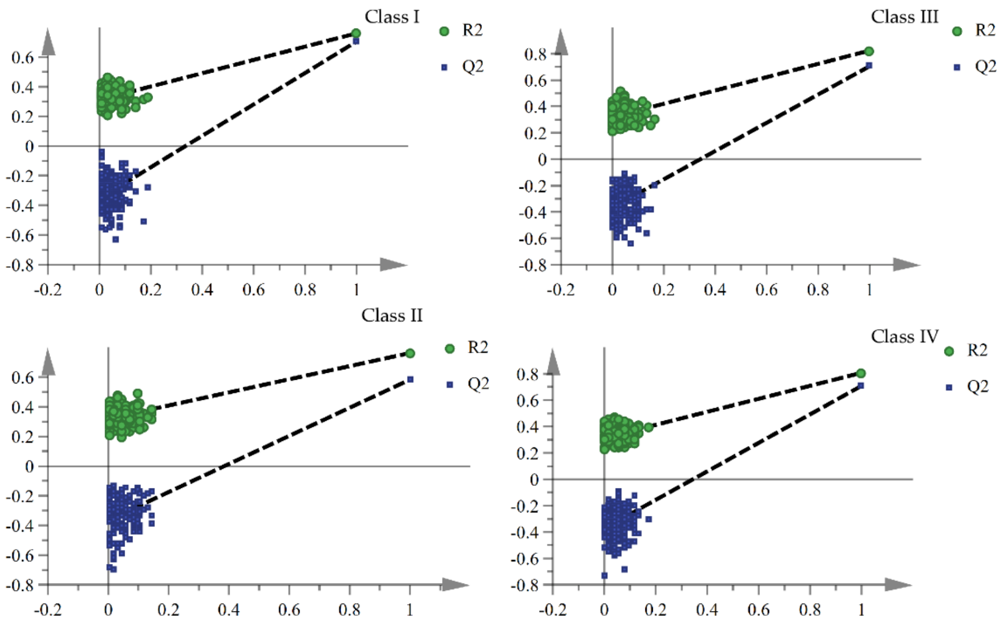


**Figure S6.** Permutation plot of the OPLS-DA of stem samples (Number of permutations = 200)

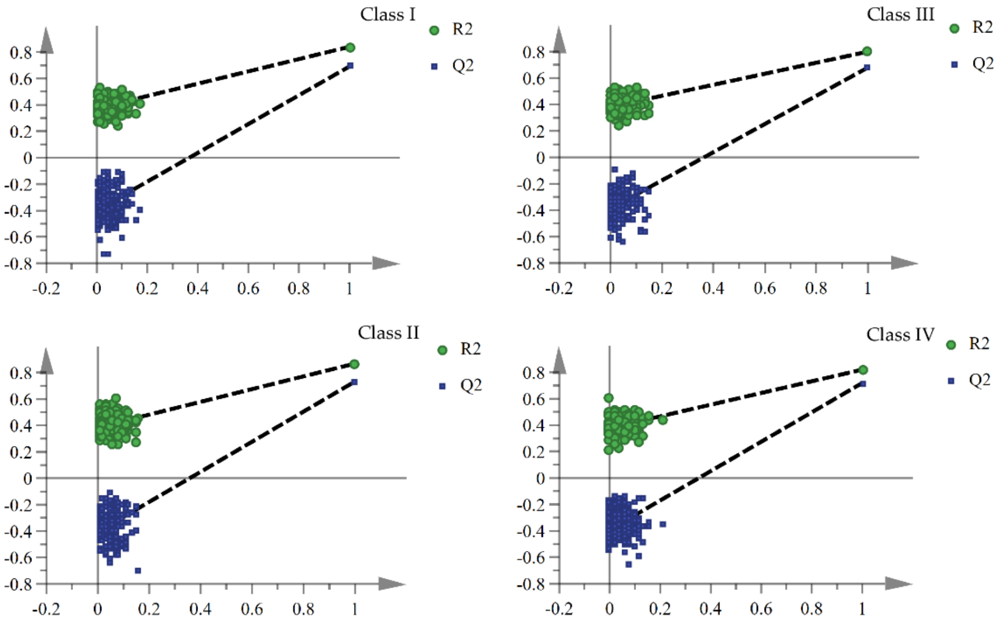


**Figure S7.** Permutation plot of the OPLS-DA of leaf samples (Number of permutations = 200)

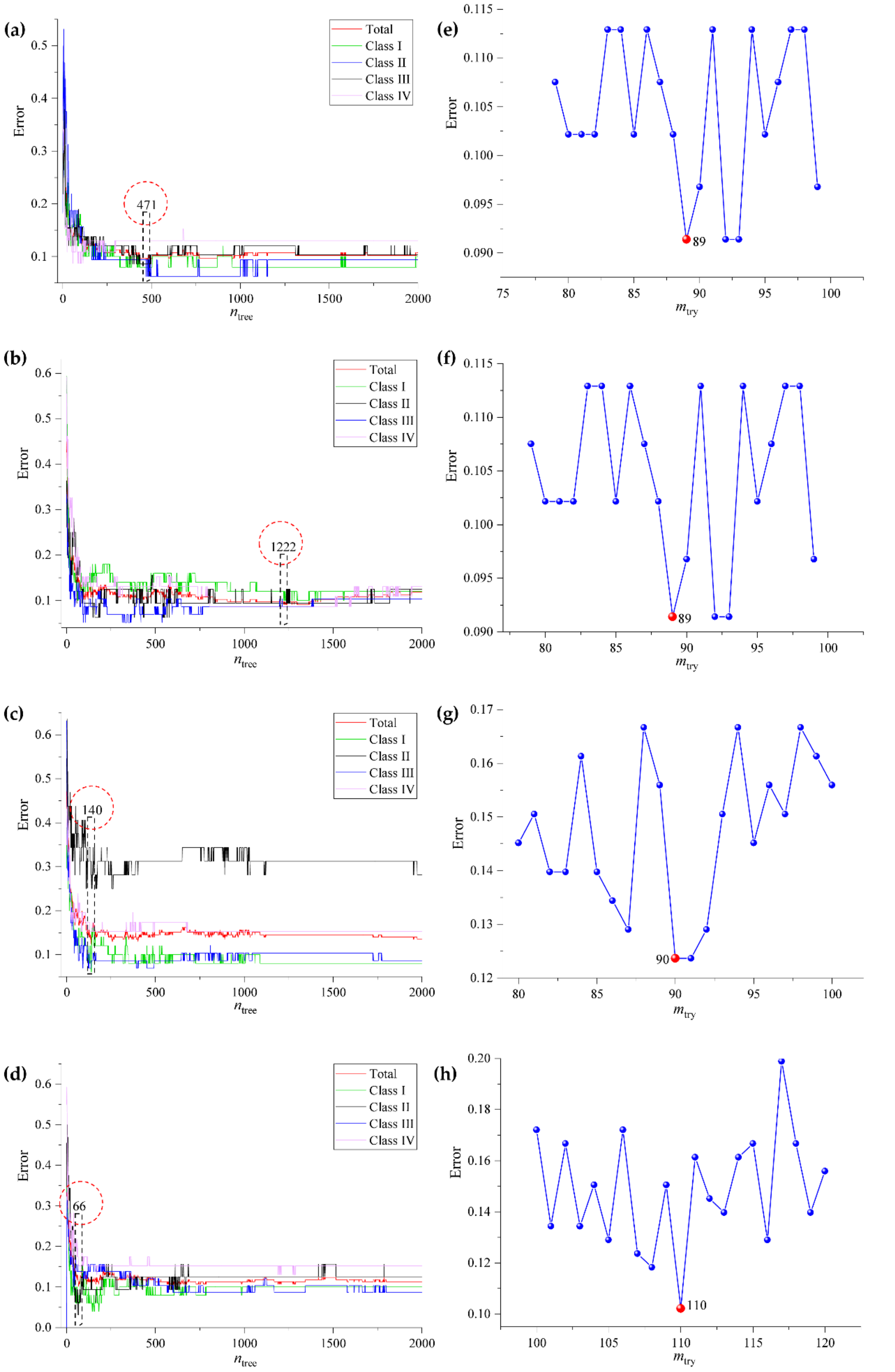


**Figure S8.** The $n_{tree}$ (a-d) and $m_{try}$ (e-h) screening of RF models based on low-level data fusion strategy (a and b = fusion data set of rhizomes and stems , c and d = fusion data set of rhizome and leaves, e and f = fusion data set of stems and leaves, g and h = fusion data set of rhizome, stem and leaves)

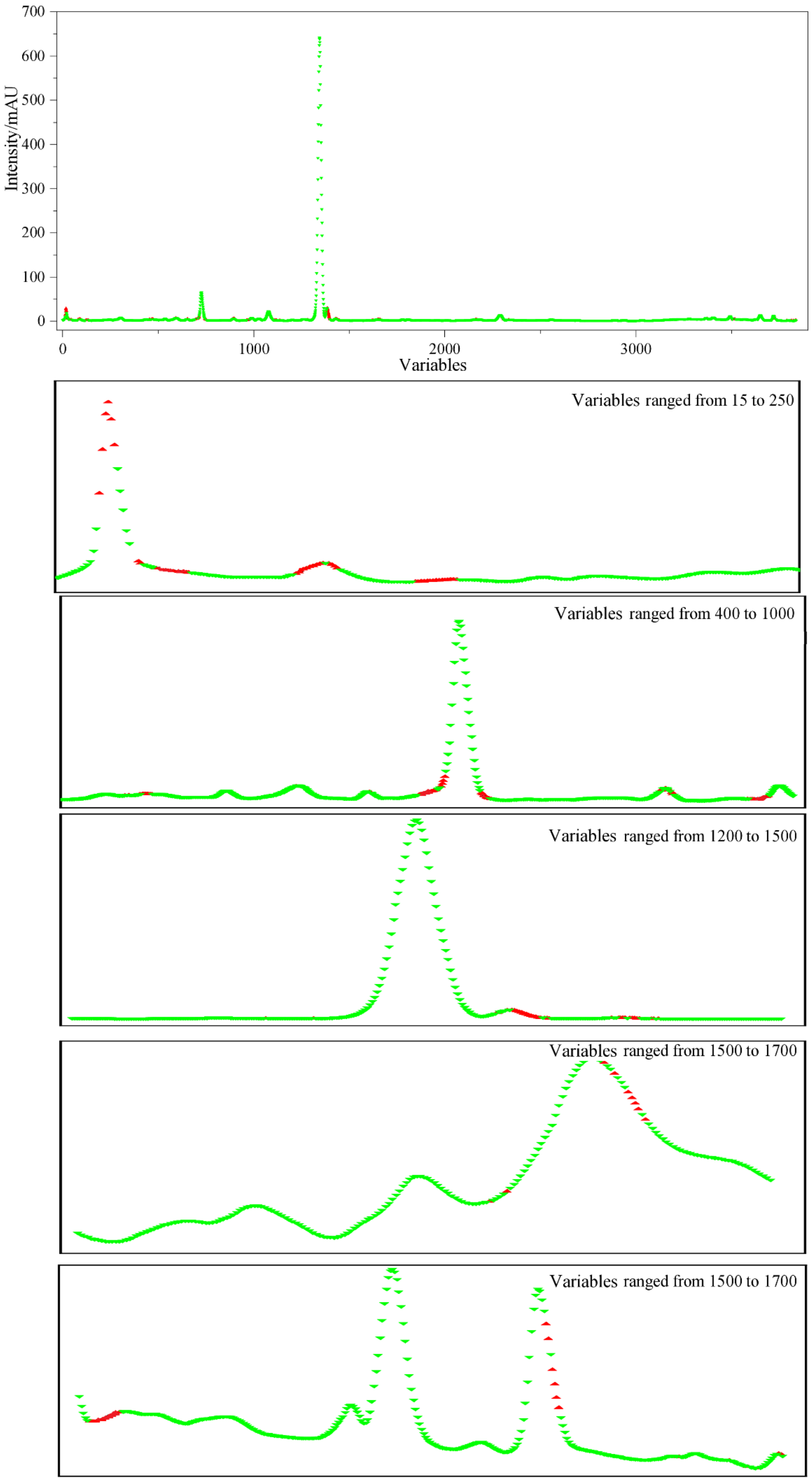


**Figure S9.** Result of variables selection of rhizome fingerprint data based on "Boruta" algorithm (red triangle = relevant features variables)

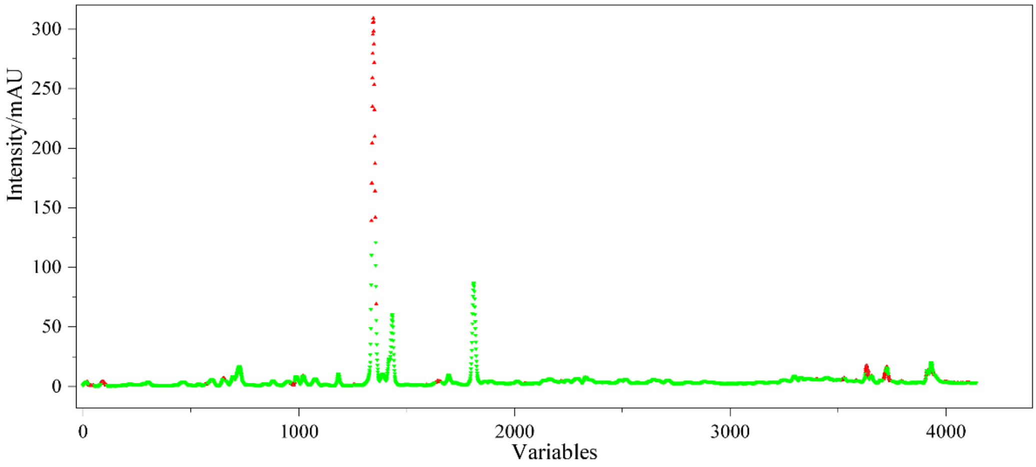


**Figure S10.** Result of variables selection of stem fingerprint data based on "Boruta" algorithm (red triangle = relevant features variables)

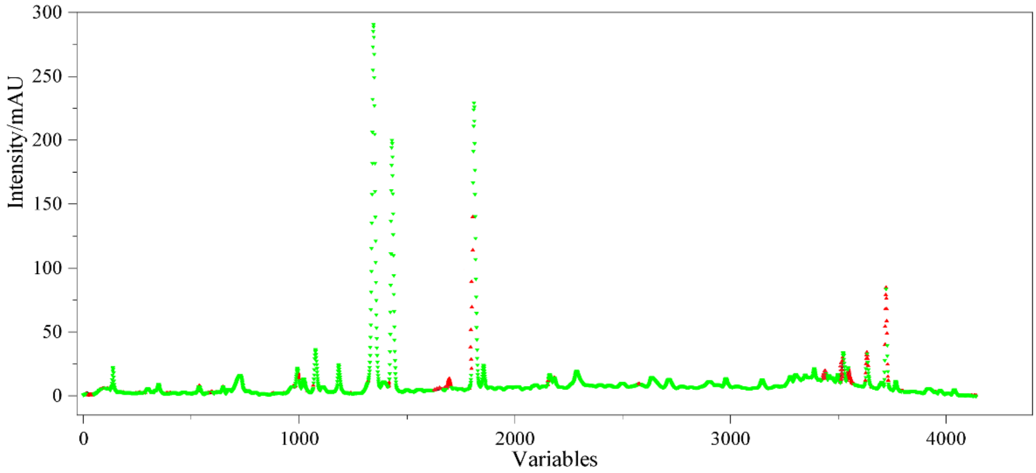


**Figure S11.** Result of variables selection of leaf fingerprint data based on "Boruta" algorithm (red triangle = relevant features variables)

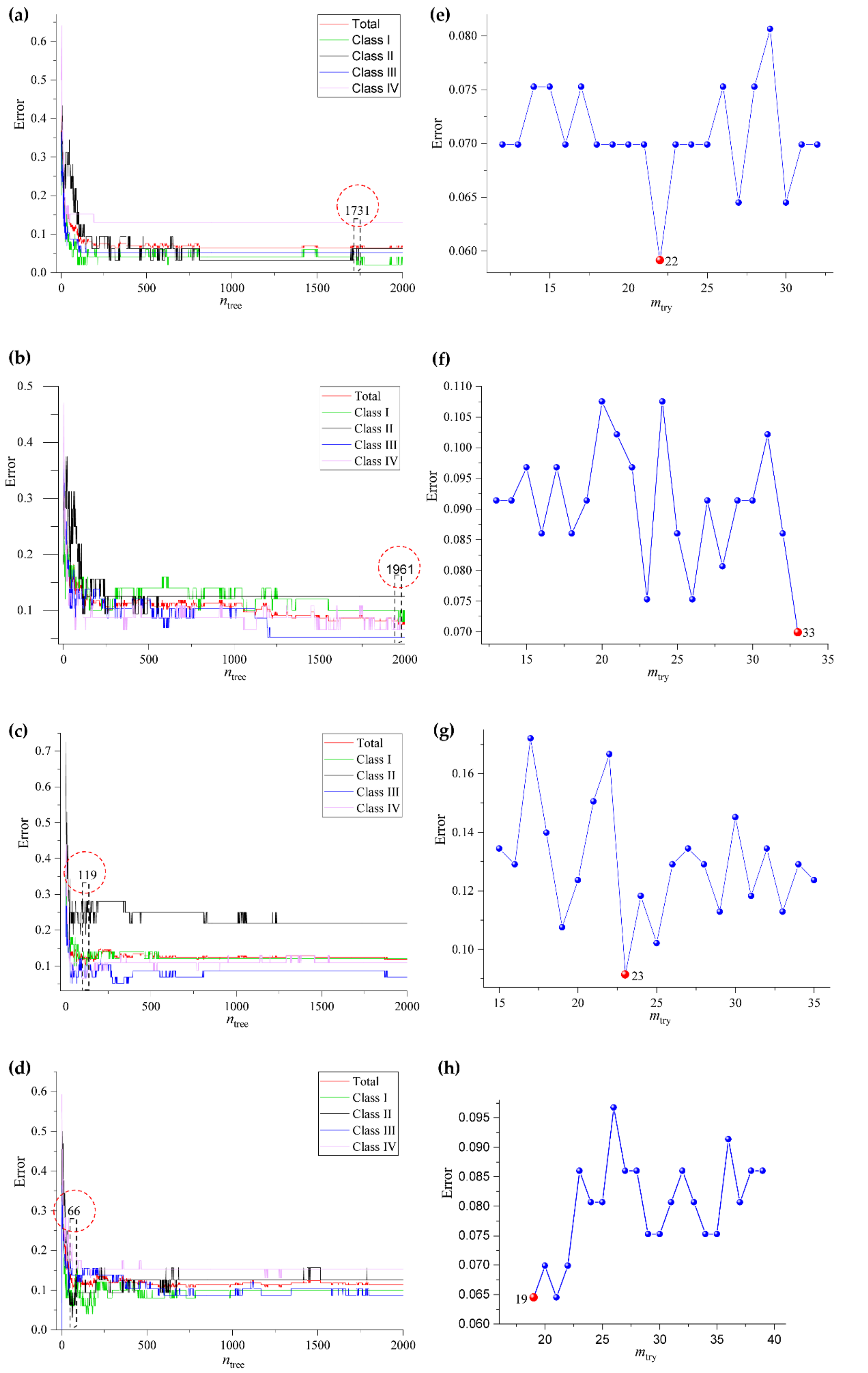


**Figure S12.** The $n_{tree}$ (a-d) and $m_{try}$ (e-h) screening of RF models based on mid-level data fusion strategy (a and b = fusion data set of rhizomes and stems, c and d = fusion data set of rhizome and leaves, e and f = fusion data set of stems and leaves, g and h = fusion data set of rhizome, stem and leaves)

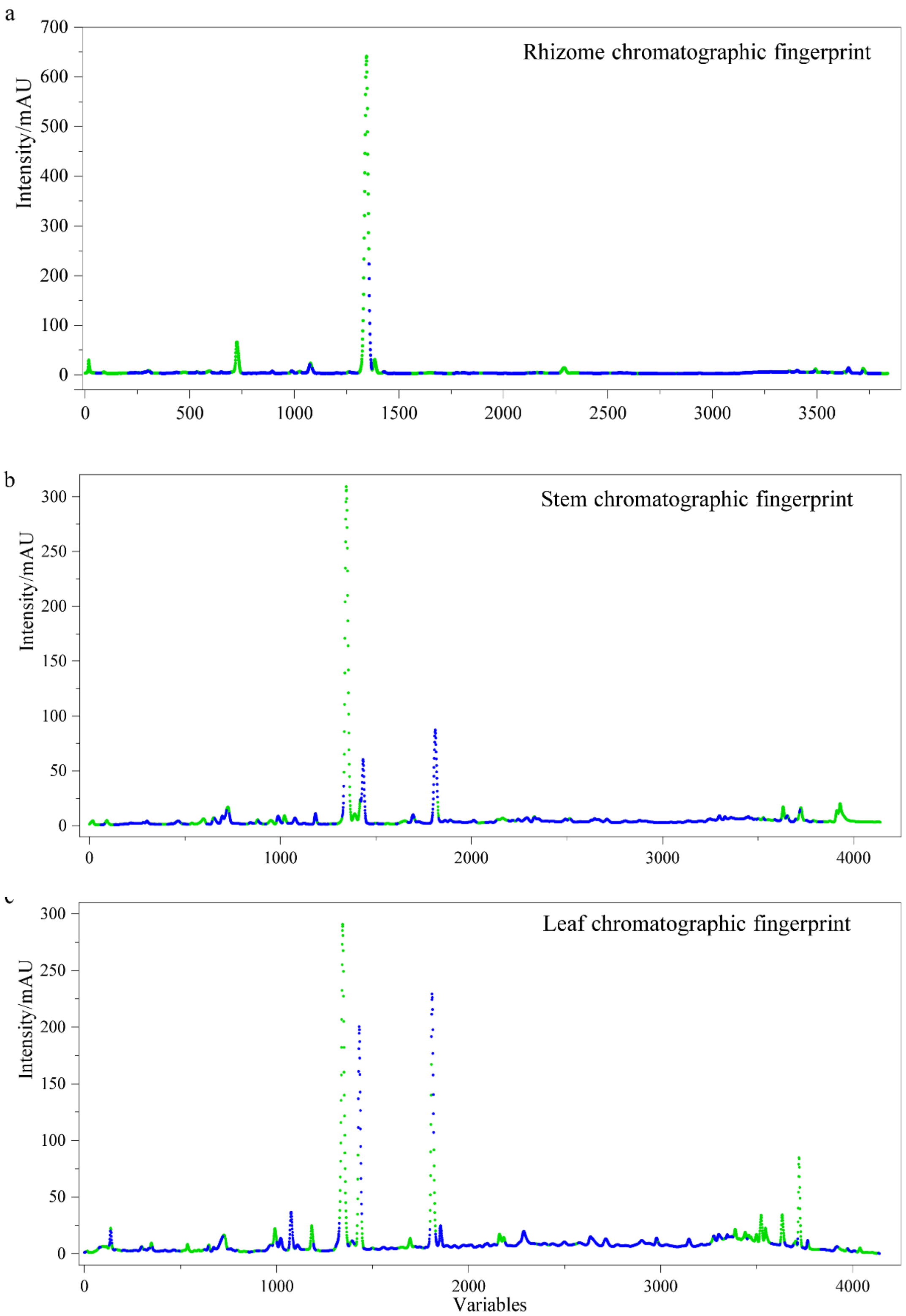


**Figure S13.** The importance variables (green circles = VIP value > 1) of OPLS-DA models of rhizomes, stems and leaves fingerprints data

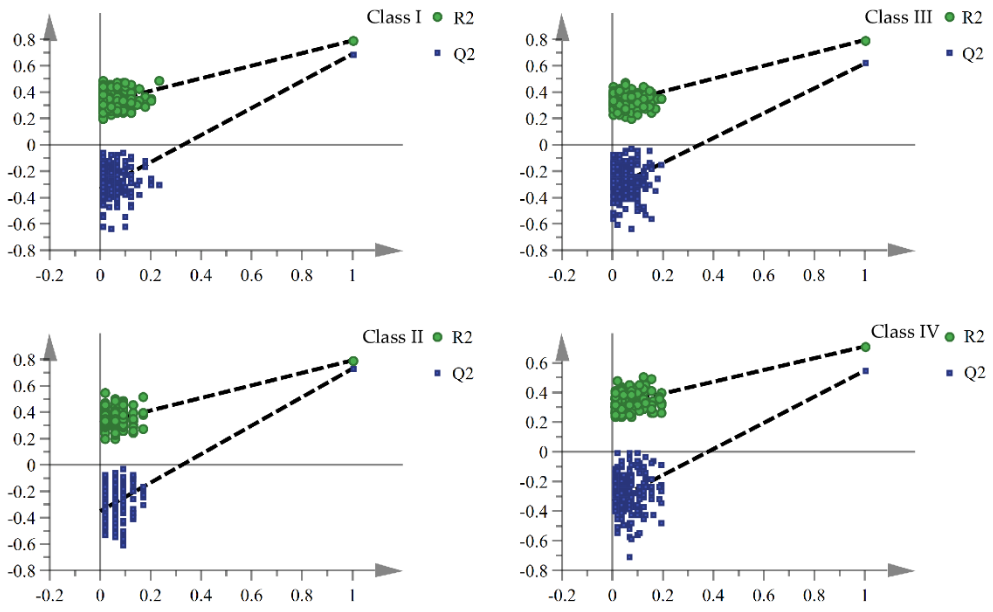


**Figure S14.** Permutation testing (200 times) of the R_OPLS-DA model

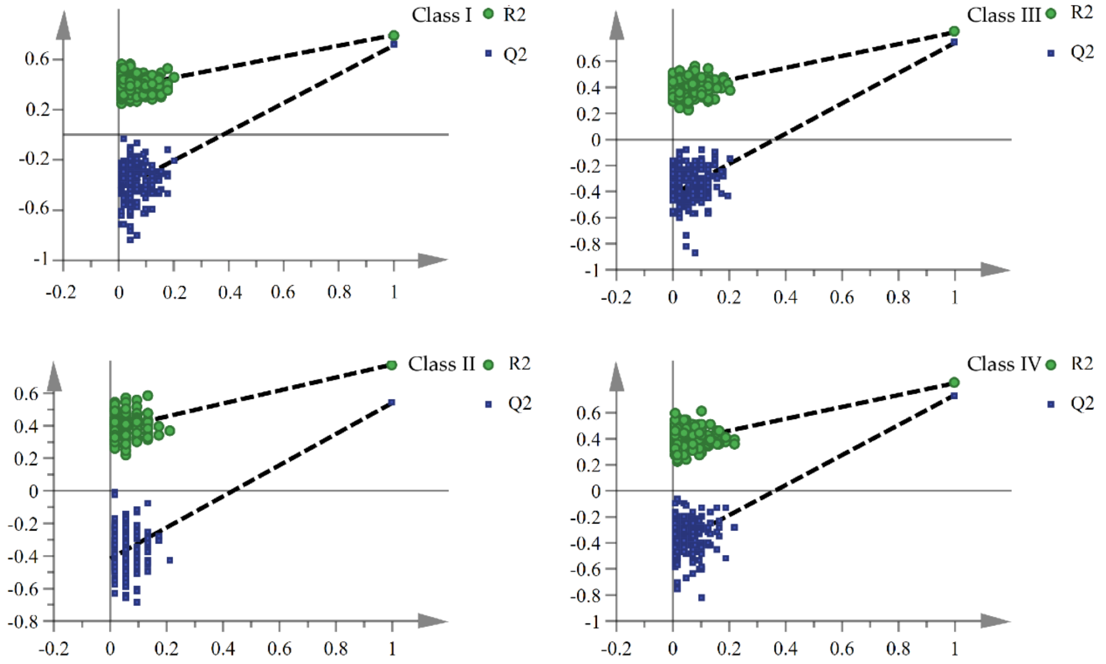


**Figure S15.** Permutation testing (200 times) of the S_OPLS-DA model

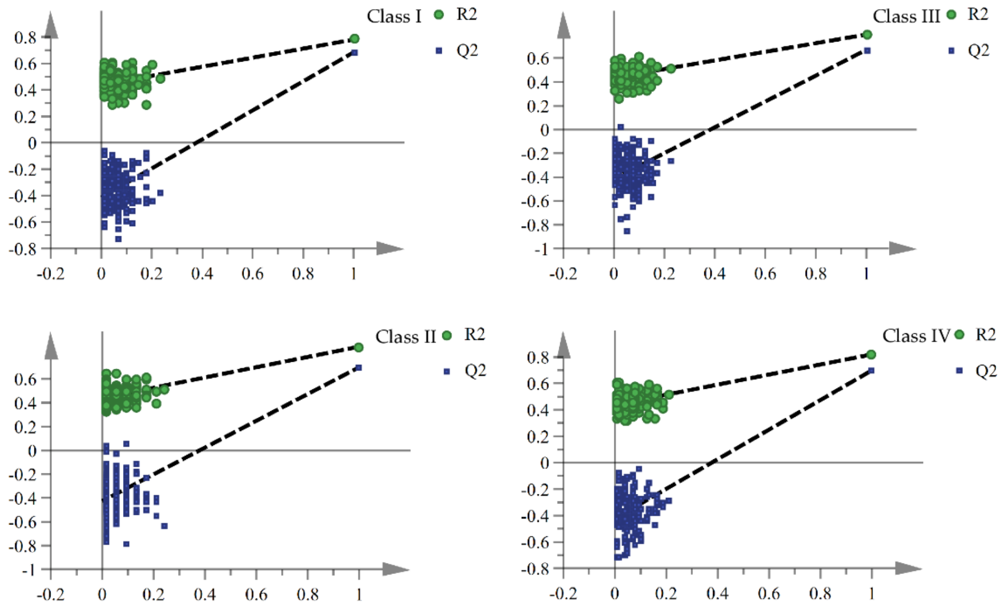


**Figure S16.** Permutation testing (200 times) of the L_OPLS-DA model

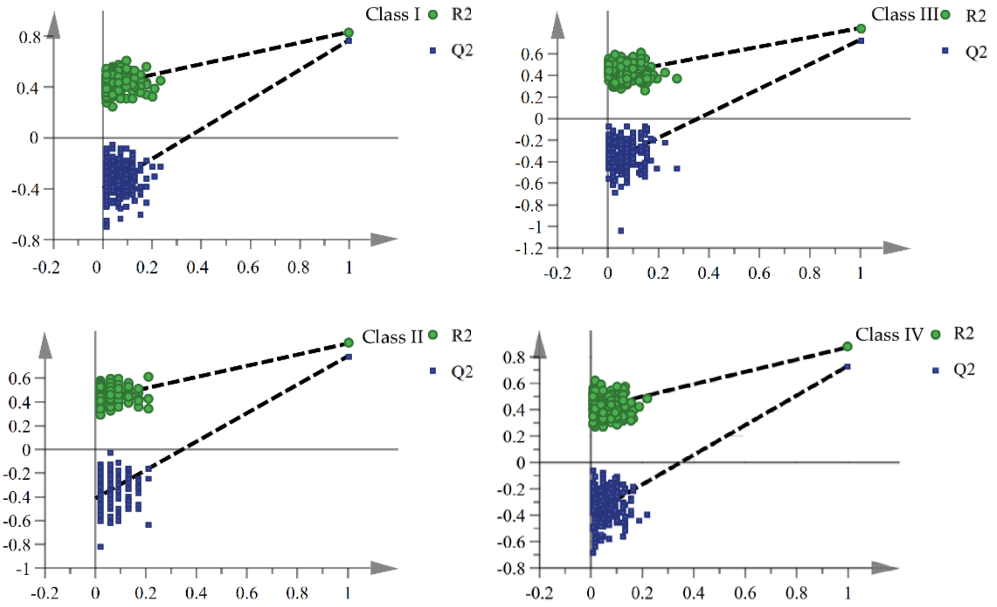


**Figure S17.** Permutation testing (200 times) of the RS_OPLS-DA model based on low-level data fusion

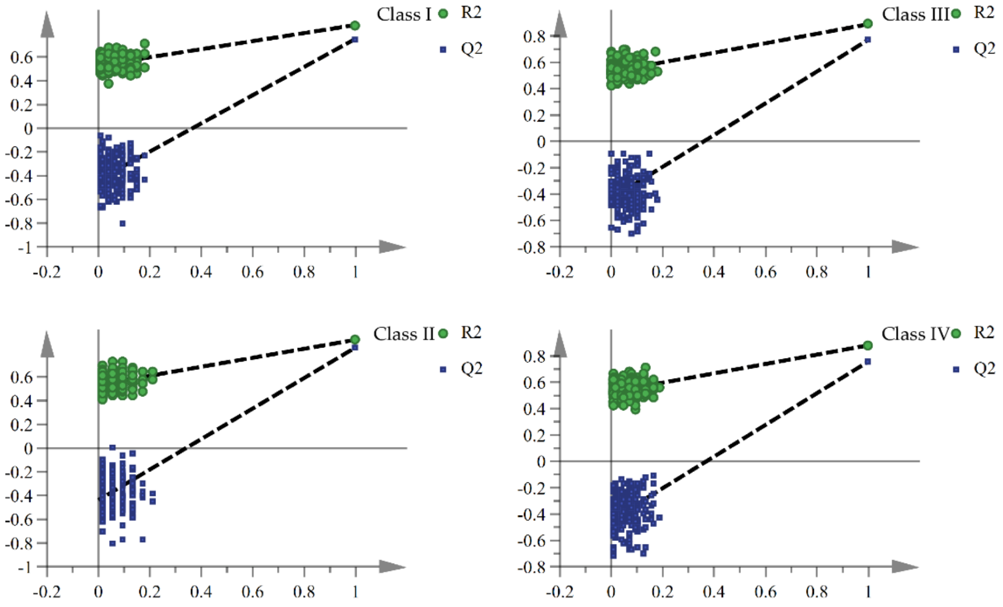


**Figure S18.** Permutation testing (200 times) of the RL_OPLS-DA model based on low-level data fusion

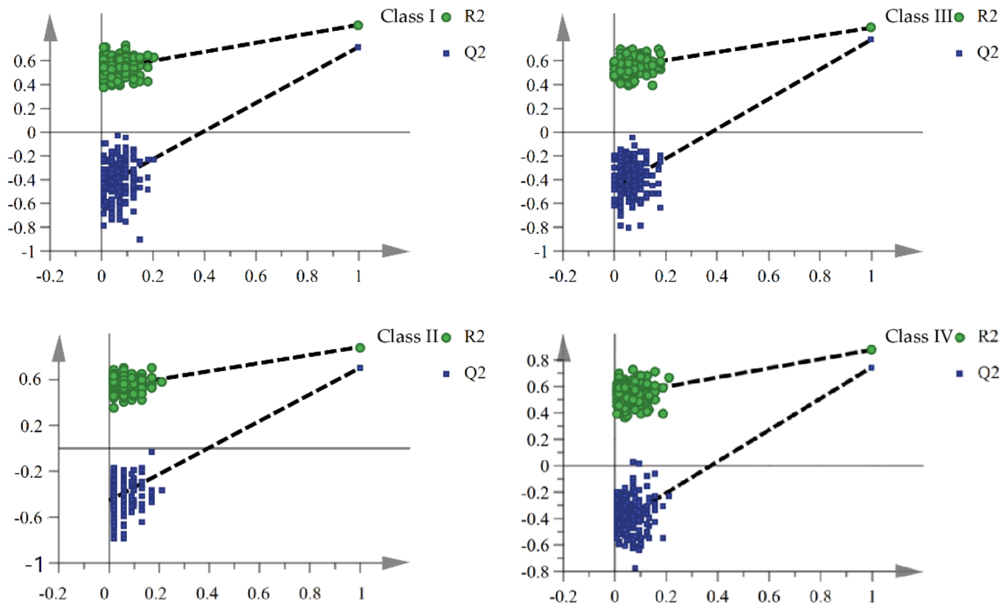


**Figure S19.** Permutation testing (200 times) of the SL_OPLS-DA model based on low-level data fusion

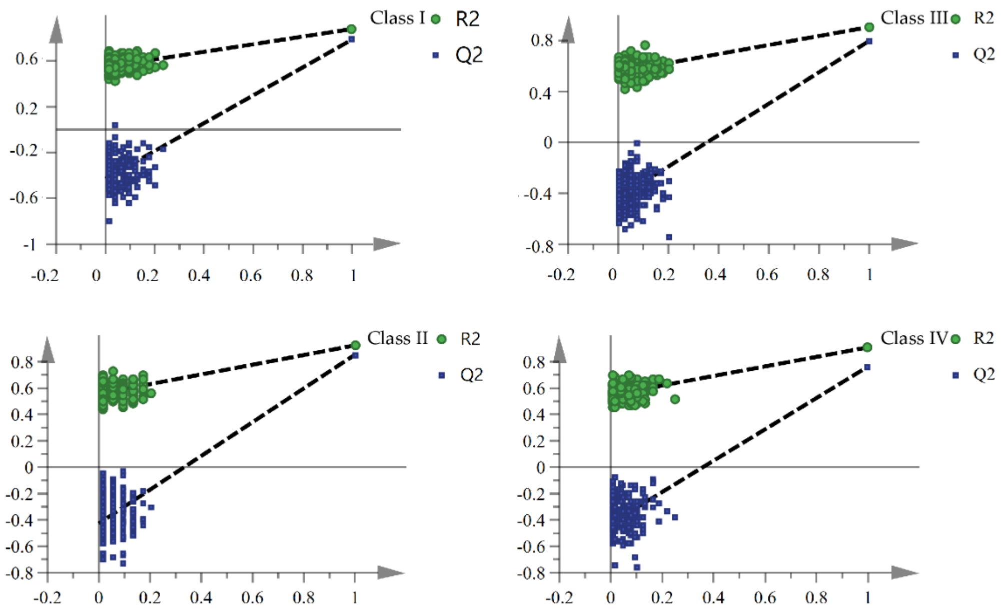


**Figure S20.** Permutation testing (200 times) of the RSL_OPLS-DA model based on low-level data fusion

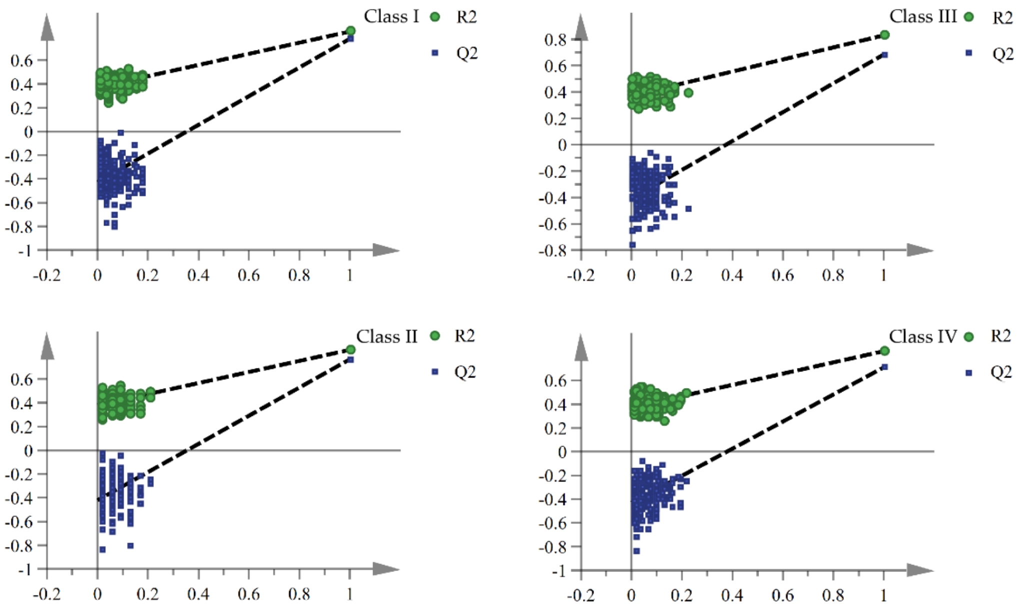


**Figure S21.** Permutation testing (200 times) of the RS_OPLS-DA model based on mid-level data fusion

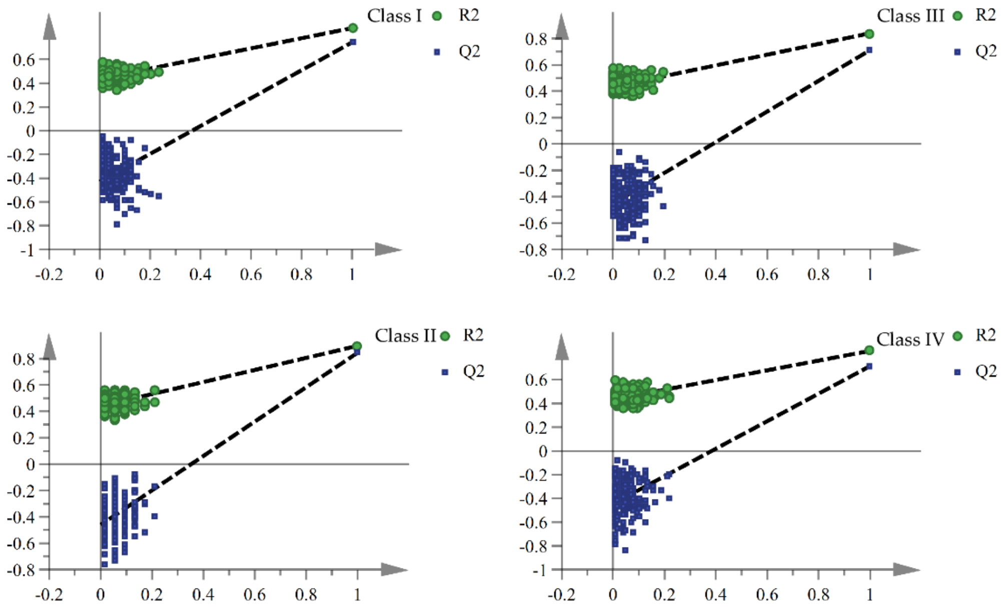


**Figure S22.** Permutation testing (200 times) of the RL_OPLS-DA model based on mid-level data fusion

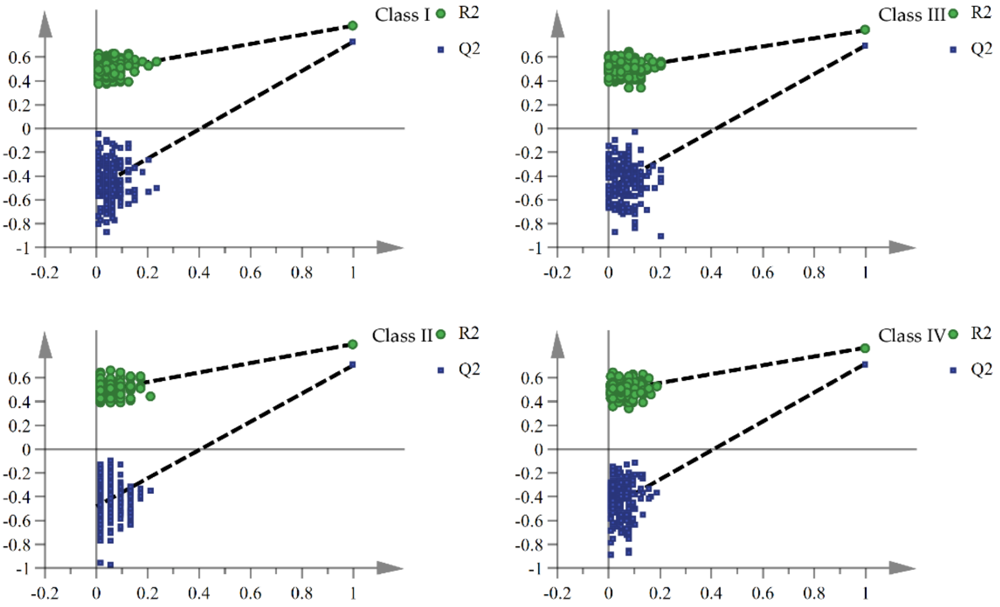


**Figure S23.** Permutation testing (200 times) of the SL_OPLS-DA model based on mid-level data fusion

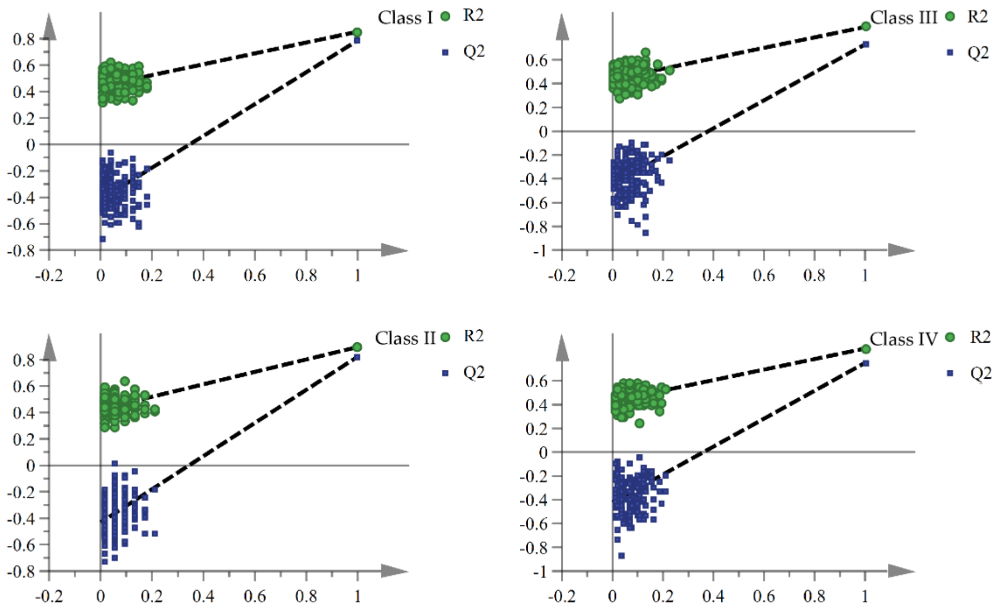


**Figure S24.** Permutation testing (200 times) of the RSL_OPLS-DA model based on mid-level data fusion

**Table S1.** The evaluation indexes for predictive power of OPLS-DA model of rhizome, stem and leaf

| Model | $R^2$ | $Q^2$ | *RMSEE* | *RMSECV* | *RMSEP* |
|---|---|---|---|---|---|
| R_OPLS-DA | 0.77 | 0.65 | 0.21 | 0.26 | 0.26 |
| S_OPLS-DA | 0.81 | 0.68 | 0.19 | 0.24 | 0.27 |
| L_OPLS-DA | 0.82 | 0.69 | 0.19 | 0.24 | 0.26 |

**Table S2.** The evaluation indexes for predictive power of OPLS-DA models based on low-level and mid-level data fusion strategies

| Model | Data fusion strategy | $R^2$ | $Q^2$ | *RMSEE* | *RMSECV* | *RMSEP* |
|---|---|---|---|---|---|---|
| RS_OPLS-DA | low-level | 0.86 | 0.75 | 0.17 | 0.21 | 0.24 |
| RL_OPLS-DA | low-level | 0.89 | 0.78 | 0.16 | 0.20 | 0.22 |
| SL_OPLS-DA | low-level | 0.89 | 0.74 | 0.15 | 0.22 | 0.23 |
| RSL_OPLS-DA | low-level | 0.90 | 0.80 | 0.14 | 0.19 | 0.22 |
| RS_OPLS-DA | mid-level | 0.84 | 0.74 | 0.18 | 0.22 | 0.24 |
| RL_OPLS-DA | mid-level | 0.86 | 0.75 | 0.17 | 0.21 | 0.24 |
| SL_OPLS-DA | mid-level | 0.86 | 0.71 | 0.17 | 0.23 | 0.24 |
| RSL_OPLS-DA | mid-level | 0.87 | 0.77 | 0.16 | 0.20 | 0.23 |
